# Diffuse, Adult-Onset Nesidioblastosis/Non-Insulinoma Pancreatogenous Hypoglycemia Syndrome (NIPHS): Review of the Literature of a Rare Cause of Hyperinsulinemic Hypoglycemia

**DOI:** 10.3390/biomedicines11061732

**Published:** 2023-06-16

**Authors:** Martin Philipp Dieterle, Ayman Husari, Sophie Nicole Prozmann, Hendrik Wiethoff, Albrecht Stenzinger, Manuel Röhrich, Uwe Pfeiffer, Wolfgang Rüdiger Kießling, Helena Engel, Harald Sourij, Thorsten Steinberg, Pascal Tomakidi, Stefan Kopf, Julia Szendroedi

**Affiliations:** 1 Division of Oral Biotechnology, Center for Dental Medicine, Medical Center—University of Freiburg, Faculty of Medicine, University of Freiburg, Hugstetterstr. 55, 79106 Freiburg, Germany; 2 Department of Orthodontics, Center for Dental Medicine, Medical Center–University of Freiburg, Faculty of Medicine, University of Freiburg, Hugstetterstr. 55, 79106 Freiburg, Germany; 3 Medical Center—University of Freiburg, Faculty of Medicine, University of Freiburg, Hugstetterstr. 55, 79106 Freiburg, Germany; 4 Institute of Pathology, University Hospital Heidelberg, 69120 Heidelberg, Germany; 5 Department of Nuclear Medicine, University Hospital Heidelberg, 69120 Heidelberg, Germany; 6 Pfalzklinikum for Psychiatry and Neurology AdÖR, Weinstr. 100, 76889 Klingenmünster, Germany; 7 Independent Researcher of Neurology and Psychiatry, 72250 Freudenstadt, Germany; 8 Cancer Immune Regulation Group, German Cancer Research Center, Im Neuenheimer Feld 280, 69120 Heidelberg, Germany; 9 Division of Endocrinology and Diabetology, Department of Internal Medicine, Medical University of Graz, 8010 Graz, Austria; 10 Interdisciplinary Metabolic Medicine Trials Unit, Medical University of Graz, 8010 Graz, Austria; 11 Department of Internal Medicine I and Clinical Chemistry, University of Heidelberg, 69120 Heidelberg, Germany

**Keywords:** nesidioblastosis, hyperinsulinism, hypoglycemia, hyperinsulinemic hypoglycemia, congenital hyperinsulinism, positron-emission tomography, insulinoma, pancreatectomy, hyperplasia

## Abstract

Differential diagnosis of hypoglycemia in the non-diabetic adult patient is complex and comprises various diseases, including endogenous hyperinsulinism caused by functional β-cell disorders. The latter is also designated as nesidioblastosis or non-insulinoma pancreatogenous hypoglycemia syndrome (NIPHS). Clinically, this rare disease presents with unspecific adrenergic and neuroglycopenic symptoms and is, therefore, often overlooked. A combination of careful clinical assessment, oral glucose tolerance testing, 72 h fasting, sectional and functional imaging, and invasive insulin measurements can lead to the correct diagnosis. Due to a lack of a pathophysiological understanding of the condition, conservative treatment options are limited and mostly ineffective. Therefore, nearly all patients currently undergo surgical resection of parts or the entire pancreas. Consequently, apart from faster diagnosis, more elaborate and less invasive treatment options are needed to relieve the patients from the dangerous and devastating symptoms. Based on a case of a 23-year-old man presenting with this disease in our department, we performed an extensive review of the medical literature dealing with this condition and herein presented a comprehensive discussion of this interesting disease, including all aspects from epidemiology to therapy.

## 1. Introduction

Hypoglycemia in an adult patient can have diverse causes. Apart from drugs such as insulin or insulin secretagogues, alcohol ingestion, critical illness (including sepsis, hepatic, renal, and cardiac failure), hormone deficiencies (e.g., adrenal or pituitary insufficiency) or endogenous overproduction of insulin and insulin-like growth factors are important to consider [1]. When evaluating apparently healthy patients, it is important to distinguish reactive/functional hypoglycemia from endogenous hyperinsulinism/hyperinsulinemic hypoglycemia [2]. The latter is defined by low blood glucose with simultaneous, inadequately high insulin/proinsulin and C-peptide levels [3].

In an adult patient, endogenous hyperinsulinism is mainly caused by insulinoma, functional β-cell disorders, insulin autoimmune hypoglycemia (Hirata’s disease), or insulin secretagogues [1]. Insulinomas are tumors arising from the β-cells of the islets of Langerhans and are usually benign. They account for approximately 90% of cases of adult-onset hyperinsulinemic hypoglycemia [4]. Hirata’s disease is caused by antibodies against insulin or the insulin receptor. The most accepted current pathophysiological hypothesis is the double-phase mechanism: in the postprandial phase, the insulin autoantibodies bind to insulin or the insulin receptor and prevent the interaction of insulin and its receptor, potentially resulting in hyperglycemia. Later, the antibodies dissociate from insulin or the receptor, enabling the proper receptor–substrate interaction, which lowers the blood glucose irrespective of the current blood glucose concentration [5,6].

Functional β-cell disorders are a group of less well-defined entities, which are also characterized by excess endogenous secretion of insulin but whose morphological features can vary considerably between individuals. Since 2005, functional β-cell disorders have frequently been reported in association with bariatric surgery, especially in patients who underwent Roux-en-Y bypass operations [7]. These disorders are often subsumed under the term “nesidioblastosis”, which originally describes the neoformation of nesidioblasts, i.e., the cells building the islets of Langerhans [8]. The scientist to first describe this condition, George F. Laidlaw, wrote in 1938:

*“…there is some evidence pointing to a diffuse or disseminated proliferation of islet cells as a possible cause of hypoglycemia. Such a diffuse proliferation of nesidioblasts would be a nesidioblastosis.”*

This description aimed at separating the findings of diffuse islet cell proliferation from localized adenomas (= nesidioblastoma/insulinoma) of the endocrine pancreas [9]. As nesidioblastosis is a morphological/histopathological term, the diagnosis can be established only in terms of the pathological evaluation of a biopsy or a surgical specimen. Clinical and biochemical findings are often described as non-insulinoma pancreatogenous hypoglycemia syndrome (NIPHS). The adult-onset form is considerably less frequent than the findings associated with congenital hyperinsulinism (CHI)/persistent hyperinsulinemic hypoglycemia of infancy and childhood (PHH). CHI/PHH is usually caused by well-known mutations in various genes associated with insulin secretion, and clinical symptoms appear within the first weeks or months after birth [10]. To separate the pathological findings in CHI/PHH from adult-onset nesidioblastosis, Anlauf and colleagues defined major and minor criteria for the latter´s diagnosis in 2005. The major criteria include microscopic, macroscopic, and immunohistochemical exclusion of an insulinoma, β-cells with hyperchromatic and enlarged nuclei and abundant clear cytoplasm, normal distribution of the various cell types (i.e., A, B, D, and PP cells) within the islets of Langerhans, as well as endocrine cells without (significant) proliferative activity. These criteria should be present in each case. Minor criteria are, however, not fulfilled by every patient and comprise enlarged islets (islet hypertrophy), a lobulated structure of the islets, an increase in the number of islets (islet hyperplasia), and macronucleoli in β-cells [11,12]. The presence of so-called ductulo–insular complexes, which describes the proximity or even budding of islets or islet cells from exocrine pancreatic ducts, is no hallmark for the diagnosis of the disease since autopsy studies have shown that at least mild forms of this finding frequently occur in healthy/asymptomatic adults [13]. This is important to know, as the term nesidioblastosis is sometimes used synonymic to ductulo–insular complexes or the scattering of single islet cells throughout the exocrine pancreatic tissue [14,15,16,17]. Confusion additionally arises from the many synonyms that are used to describe this disease. The terms endocrine cell dysplasia, nesidiodysplasia, multifocal ductulo–insular proliferation, islet cell adenomatosis, islet hyperplasia, and islet cell hypertrophy are frequently found in the literature [14,18,19,20].

In the following review, we describe and discuss all relevant aspects of adult-onset nesidioblastosis/NIPHS and related morphological findings, including epidemiology, pathophysiology, clinical findings, and principles of therapy. Additionally, we recently presented a case of a 23-year-old man with this extremely rare disease in a separate Case Report, which beautifully illustrates many facets of the diagnostic process and potential pitfalls (see Dieterle et al. 2023, accepted for publication in *Biomedicines*).

## 2. Methodology of the Literature Research and Limitations

We performed a literature research to find out more about this intriguing disease and to describe interesting associations of islet cell hyperplasia/nesidioblastosis with other diseases. Our semi-systematic research strategy included searches for “nesidioblastosis”, “islet [cell] hyperplasia”, “endocrine cell dysplasia”, “islet [cell] hypertrophy”, “β OR B OR beta cell hyperplasia”, “hyperinsulinemic hypoglycemia”, “islet [cell] adenomatosis”, “nesidiodysplasia”, “multifocal ductulo-insular [ductuloinsular] complexes”, “α OR alpha cell hyperplasia”, “D OR somatostatin cell hyperplasia”, and “PP OR pancreatic polypeptide cell hyperplasia”, in Medline until May 2022. The expressions in the next step also combined with “adult” to exclude the pediatric population, where much more literature is available. A literature research with the corresponding MeSH terms was also performed. Additionally, the reference sections of the retrieved papers were systematically screened for further publications. In total, 460 publications of interest were found. Unfortunately, in one-third of the publications, full texts were not available. Nonetheless, we provide a comprehensive overview of the main findings of these research papers in Supplementary Table S1. This is, to the best of our knowledge, the most complete presentation of such cases. Where available, we documented the year of publication, the number of cases, special features of the case (including age and sex of the patients), pathological features of the pancreas, clinical symptoms/laboratory findings, therapy, and a (personal) evaluation of the case. Missing full texts are indicated by an asterisk * in Supplementary Table S1. When the literature was found only as a reference in another original paper, we describe this in the “literature” section of Supplementary Table S1. The references for Supplementary Table S1 can be found in the reference section [3,5,7,11,13,14,15,16,17,18,19,20,21,22,23,24,25,26,27,28,29,30,31,32,33,34,35,36,37,38,39,40,41,42,43,44,45,46,47,48,49,50,51,52,53,54,55,56,57,58,59,60,61,62,63,64,65,66,67,68,69,70,71,72,73,74,75,76,77,78,79,80,81,82,83,84,85,86,87,88,89,90,91,92,93,94,95,96,97,98,99,100,101,102,103,104,105,106,107,108,109,110,111,112,113,114,115,116,117,118,119,120,121,122,123,124,125,126,127,128,129,130,131,132,133,134,135,136,137,138,139,140,141,142,143,144,145,146,147,148,149,150,151,152,153,154,155,156,157,158,159,160,161,162,163,164,165,166,167,168,169,170,171,172,173,174,175,176,177,178,179,180,181,182,183,184,185,186,187,188,189,190,191,192,193,194,195,196,197,198,199,200,201,202,203,204,205,206,207,208,209,210,211,212,213,214,215,216,217,218,219,220,221,222,223,224,225,226,227,228,229,230,231,232,233,234,235,236,237,238,239,240,241,242,243,244,245,246,247,248,249,250,251,252,253,254,255,256,257,258,259,260,261,262,263,264,265,266,267,268,269,270,271,272,273,274,275,276,277,278,279,280,281,282,283,284,285,286,287,288,289,290,291,292,293,294,295,296,297,298,299,300,301,302,303,304,305,306,307,308,309,310,311,312,313,314,315,316,317,318]. In total, we collected information from approximately 330 research papers, which may contribute to a deeper understanding of the disease spectrum of adult-onset nesidioblastosis and islet cell hyperplasia.

However, there are some potential limitations of our literature research. Controversies about post-gastric bypass surgery hyperinsulinemic hypoglycemia/nesidioblastosis make it difficult to classify these patients and distinguish them from late dumping syndrome/reactive (hyperinsulinemic) hypoglycemia (see below). Additionally, in the last years, pancreatectomies to treat these patients have become more and more uncommon due to the considerable morbidity and mortality, which reduces the number of cases with histopathological diagnoses.

Only cases with definitive histopathology and some illustrative cases with clinical presentations highly suspicious of underlying nesidioblastosis/islet cell hyperplasia were included in Supplementary Table S1. Patients diagnosed with multiple endocrine neoplasia type 1 (MEN1), Zollinger–Ellison syndrome, Verner–Morrison syndrome, or islet cell adenomatosis were not studied separately since islet cell hyperplasia is a relatively common finding in this collective but is not always reported separately. Therefore, only cases where islet cell hyperplasia/nesidioblastosis is explicitly mentioned in the publications were included. The differentiation between pancreatic microadenoma/microadenomatosis (e.g., in the context of MEN1) and nesidioblastosis is sometimes arbitrary in the literature (adenoma is mostly defined as consisting of only one cell type and/or to be encapsulated). Again, only cases where islet cell hyperplasia/nesidioblastosis is explicitly mentioned in the publications were included. Islet cell hyperplasia is also sometimes found in chronic pancreatitis, areas of pancreatic fibrosis (e.g., in Cystic fibrosis), anabolic-androgenic steroid use, development of type II diabetes (at least in animal models), or while taking several drugs [65,319,320,321,322,323]. Cases comprising these conditions were only included in Supplementary Table S1 when islet cell hyperplasia/nesidioblastosis was a major characterizing feature of the patient, if hyperinsulinemic hypoglycemia or symptoms associated with hypoglycemia were present, or if the histological changes could also be attributed to other findings in the patients. Imaging studies for insulinoma detection, especially emerging methods in the field of nuclear medicine, often incidentally detect some cases of adult-onset nesidioblastosis [324,325]. Although many imaging studies are included in Supplementary Table S1, we cannot exclude that some of these studies have been overlooked since the nuclear medicine literature has not been explicitly searched for (random) detection of islet cell hyperplasia/nesidioblastosis. Some cases of adult-onset nesidioblastosis/NIPHS might also have been reported in the context of families with defined mutations (“familial hyperinsulinism”) since sometimes hyperinsulinism in adults is incidentally detected when an offspring presents with PHH/CHI. The separating line between PHH/CHI with a defined mutation and adult-onset nesidioblastosis/NIPHS with the same mutation and a clinical presentation at higher age is thus not always clear, and our literature research might have missed such cases. Some full texts and some citations found in the reference section of other publications were not available. Therefore, sometimes it was not possible to decide whether it was really a case of adult nesidioblastosis. The age at which to classify the cases as an adult is also ambiguous in the literature (see discussion below) [247]. Other papers did not fully report all demographic data to adequately classify the case (e.g., no age or gender was reported). Equivocal cases are described as such in the “Evaluation” column of Supplementary Table S1. The literature search via Medline might also be biased towards more recent literature since older publications are systematically underrepresented in the database. Whenever possible, however, original publications (dating back to 1925) were included.

## 3. History, Histopathological, and Clinical Definition of Hyperinsulinemic Hypoglycemia and Nesidioblastosis/Islet Cell Hyperplasia

Idiopathic/sporadic adult-onset nesidioblastosis with hyperinsulinemic hypoglycemia is a rare disease that occurs much less frequently than insulinoma in the adult population (see Section 6 below). Seale Harris first proposed hyperinsulinism as a clinical syndrome in 1924 [326]. The first cases of a (malignant) insulinoma were described not much later [327]. However, it is not entirely clear when the first unequivocal case of adult-onset nesidioblastosis was described in the medical literature. Some authors claim that a report by Bradley and colleagues in 1976 might be the first case [74]. Our literature research suggests that hyperplasia of the islets of Langerhans has been recognized long before. The clinical correlation to hyperinsulinemic hypoglycemia is, however, not that clear. In 1925, Lang reported nodular hyperplasia of islets (also described as adenomatosis), which could be a description of what is known today as diffuse islet cell hyperplasia [31]. As we could not retrieve the original manuscript by Lang, it is, unfortunately, unclear if there were any symptoms related to this finding and if the patient was an adult. Six years later, John reported a patient with insulin-dependent diabetes, hyperthyroidism, cirrhosis of the liver, liver/gallbladder carcinoma, and interstitial pancreatitis [31]. Insulin had to be discontinued in this patient due to hypoglycemia. The observed coexistence of hypertrophy and atrophy of the islets of Langerhans could, however, also be a consequence of pancreatitis. Hypertrophic and hyperplastic islets have been repeatedly described in this context [328]. The hypoglycemia could thus result from hepatic insufficiency and is not necessarily a consequence of endogenous hyperinsulinism.

In 1944, Frantz described different cases of insulin-producing neoplasms, adenomas, and adenomatosis. Among this cohort, there were 11 cases of hypertrophy and/or hyperplasia of the islets of Langerhans without any evidence of coexisting neoplasia [31]. Hypoglycemia was reported for all patients, and three of them underwent partial pancreatectomy. The symptoms were not relieved by the surgical interventions. From our point of view, these cases might be the first genuine descriptions of adult-onset, diffuse nesidioblastosis with clinically detected hypoglycemia. In the subsequent years between 1944 and the case presented by Bradley in 1976, other authors, such as Vance et al., reported patients with hypoglycemia symptoms and concomitant islet cell hyperplasia. The latter cases were found in a family suffering from MEN1 [172]. Of interest, Stefanini and colleagues reviewed approximately 130 cases of adult patients who presented with hypoglycemia and showed islet cell hyperplasia upon surgery or autopsy [216]. They reported that symptom onset preceded diagnosis by 4.5 years on average. A total of 122 of the 148 cases reported (among them 16 children) underwent surgery, and symptom control was achieved in approximately 70%. These cases are especially interesting since, at that time, hyperplasia of the islets of Langerhans as a cause of CHI was widely accepted, whereas adult patients with organic hyperinsulinism were thought to suffer exclusively from insulinomas. To the best of our knowledge, the publication by Stefanini and colleagues represents the oldest and largest series of patients with islet cell hyperplasia, which also contains otherwise unpublished cases from personal communications with physicians from all around the world. It can be assumed that these cases would today be diagnosed as having NIPHS or diffuse, adult-onset nesidioblastosis.

The definition of adult-onset nesidioblastosis still leads to confusion among physicians today. Some assume that hyperinsulinemic hypoglycemia is always related to β-cell neoplasia, namely insulinoma, and that cases diagnosed as nesidioblastosis or islet cell hyperplasia represent overlooked, small tumors/insulinomas [329,330]. However, thorough histopathological work-up of many pancreata from patients diagnosed with nesidioblastosis did not lead to the detection of occult insulinomas [11].

As described above, the terms nesidioblastosis, endocrine cell dysplasia, nesidiodysplasia, multifocal ductulo–insular proliferation, islet cell adenomatosis, islet hyperplasia, and islet cell hypertrophy are frequently used as synonyms to describe roughly the same findings, which sometimes complicates communication among experts. From a histopathological point of view, nesidioblastosis is a questionable denomination since the initial definition by Laidlaw does not adequately fit the current diagnostic criteria for adult-onset nesidioblastosis as proposed by Anlauf et al., which rely on the findings in a relatively big number of patients with hyperinsulinemic hypoglycemia [11]. While Laidlaw underscores the diffuse or disseminated proliferation of islet cells, the criteria by Anlauf and colleagues stress the existence of endocrine cells without significant proliferative activity, e.g., represented by a low Ki-67 index. Yakovac proposed the following definition in 1971 (albeit for pediatric patients): *“…continuous or continual differentiation of insulin-producing β-cells from any or all divisions of the ductular system of the exocrine pancreas. […] However, it is in the additional differentiation solely of abundant, tiny clusters of β-cells and their commingling, in diffuse and random fashion, about or within acinar parenchymal elements, that the term “beta cell nesidioblastosis” signifies a condition of pathophysiologic importance.”* [331]. However, nesidioblastosis according to these definitions, i.e., proliferation of endocrine cells within the adult pancreas, as represented by the neo-formation of islets from pancreatic ducts (“ductulo-insular complexes”), as well as scattered proliferation/distribution of small groups of endocrine cells within the exocrine parenchyma of the pancreas or focal islet cell hyperplasia, are also found in autopsy studies of asymptomatic adults [13,236,332]. Since one major diagnostic criterion is the presence of enlarged, hyperchromatic nuclei in β-cells, the terms “nesidiodysplasia”, ” islet dysplasia”, or “islet cell atypia” would fit better because these properties are also found in atypic cells, and the islets themselves possess histoarchitecture disturbances [333]. As β-cells are explicitly mentioned, this eliminates the possibility of classifying similar morphological findings of other pancreatic endocrine cell types under the term nesidioblastosis and would also limit its clinical application to patients suffering from hyperinsulinemic hypoglycemia.

Regular patterning and spatial distribution of the different endocrine cell types (A/alpha/α-cells (glucagon-secreting), B/beta/β-cells (insulin-secreting), D-cells (somatostatin-secreting), PP-cells (pancreatic polypeptide secreting)) additionally excludes findings, where one cell type within abnormal endocrine pancreata dominates (e.g., in genuine insulinomatosis with a clear predominance of β-cells or the various cases of pancreatic polypeptide (PP) or alpha/A/glucagon cell hyperplasia) [32,39,74,245]. The latter findings can, however, produce clinically detectable symptoms associated with excess hormone synthesis and release, although not necessarily related to insulin. These morphologies are sometimes reported as α-cell- or PP-cell-nesidioblastosis or -hyperplasia (see below), which further complicates the differentiation of these findings.

Exclusion of an insulinoma by means of macroscopic, microscopic, and immunohistochemical evaluation of the tissue is the last major criterion to diagnose diffuse, adult-onset nesidioblastosis. From our point of view, this is problematic because many patients have been reported that suffered from pathologically detectable insulinoma and concomitant islet cell hyperplasia/nesidioblastosis (also termed “background nesidioblastosis”) [16,58,75,109,231,235,283,285,286,316,317]. One patient was even reported to have an insulinoma relapse, which was possibly related to the coexistence of islet cell hyperplasia/nesidioblastosis [231]. The finding of coexisting insulinoma and changes in the morphology and probably also a function of the remaining islets of Langerhans is thus no rarity and not necessarily mutually exclusive.

Another important point is the differentiation of diffuse and focal adult nesidioblastosis. While focal forms of hyperinsulinemic hypoglycemia account for roughly 40% of cases in children/infants, focal adult nesidioblastosis is extremely rare, and only 10 cases have been described in the medical literature [12,55,111,151,159,203,210,225,250]. All other forms are described as diffuse, adult-onset nesidioblastosis since the morphological findings are distributed throughout (nearly) the entire pancreas.

Concerning the minor diagnostic criteria, islet hyperplasia is most often (and sometimes the only feature) reported in the literature, meaning an increase in the number of islets per area. Winstock and co-workers even suggested replacing the term nesidioblastosis with islet cell hyperplasia to create a terminology that includes nesidioblastosis, islet hypertrophy, septal islets, islet dysplasia, and adenomatosis. However, especially in patients suffering from post-bariatric surgery hyperinsulinemic hypoglycemia, the presence of islet hyperplasia has been questioned [95]. Moreover, Goudswaard and colleagues convincingly showed that the total endocrine area could not distinguish nesidioblastosis-hyperinsulinism from normal adult pancreata [329]. They proposed that the impression of islet hyperplasia arose when immunohistochemical staining was introduced in the evaluation of pancreata from patients with neuroendocrine disorders, and endocrine cells were suddenly better recognized than by standard hematoxylin and eosin (HE) staining. Therefore, they also questioned the existence of the pathologic entity “nesidioblastosis” in non-bariatric patients.

Islet hypertrophy, the enlargement of islets, is also frequently reported, while islets’ diameters > 300 µm are regarded as pathologic [12]. Islet hyperplasia and hypertrophy, as well as a lobulated islet structure or macronucleoli of β-cells, however, vary from patient to patient, rendering a standardized evaluation difficult [281]. Klöppel et al. concluded that approximately 2/3 of patients exhibit pancreata fulfilling all diagnostic criteria, whereas 1/3 of the specimens could also be classified as “normal” [12].

Some authors also reported peliosis, i.e., vascular ectasia, within the islets of Langerhans in patients (up to 50% in nesidioblastotic pancreata vs. 12% in normal controls) suffering from hyperinsulinemic hypoglycemia [137]. The significance of this finding is, however, not entirely clear.

From a clinical point of view, morphological findings declared as nesidioblastosis or islet cell hyperplasia are found in different diseases (see also next section). As in the case recently presented by the authors of this review (see Dieterle et al. 2023; accepted for publication in *Biomedicines*), there sometimes exists a good correlation of clinical findings, i.e., endogenous hyperinsulinism, with pathological findings. Then, how should these symptoms be named clinically? Non-insulinoma pancreatogenous hyperinsulinemic hypoglycemia syndrome (NIPHS) was introduced by Service and colleagues in 1999 [45], which is a good description of the etiology and clinics of hyperinsulinemic hypoglycemia associated with a functional β-cell disorder. However, the original definition of NIPHS relies on several criteria, including postprandial neuroglycopenic symptoms with concomitant detectable hypoglycemia, a negative 72 h fasting test, negative preoperative imaging studies, and a positive selective arterial calcium stimulation test with hepatic venous sampling (SACS). These criteria are not fulfilled by every patient, as some (such as the case presented by the authors of this review) have a positive fasting test without insulinoma, show exercise-induced hypoglycemia, or other additional features [71]. The histological appearance of these patients is, however, not different from NIPHS patients, strictly fulfilling the diagnostic criteria. Some authors also diagnose a nesidioblastosis clinically, which is impossible by definition [117].

To overcome the shortcomings in the current reporting of such cases, we propose a standardized work-up of the clinical (see below) and, where available, histopathological features (according to Anlauf et al., 2005, Raffel et al. 2007, or Klöppel et al. 2008 [11,12,93]) that include the following points (also see Figure 3 of our Case Report; Dieterle et al. 2023; accepted for publication in *Biomedicines*):

(1) Evaluation of general hypoglycemia symptoms and their situational occurrence (i.e., postprandial, fasting, spontaneous, exercise-induced);
(2) Results of a 4 h (–6 h) oral glucose tolerance test (OGTT);
(3) Results of a 72 h fasting test;
(4) Results of conventional imaging studies (CT, MRI);
(5) Optional (might replace the next point in the future): results of functional imaging studies (where available, e.g., ^68^Ga-DOTA-Exendin-4 PET/CT or Somatostatin-receptor scintigraphy);
(6) SACS with proof of an insulin gradient (might also be useful to define the extent of pancreatic resection if surgery is planned);
(7) Exclusion of other conditions (e.g., Hirata´s disease, insulin secretagogues, etc.).

If the clinical findings prove hyperinsulinemic hypoglycemia and conventional imaging studies are negative; SACS shows an increased, non-localized insulin response; and/or functional imaging studies suggest diffuse tracer enrichment compatible with a functional β-cell disorder, we think that the clinical diagnosis of a NIPHS is justified, even if the fasting test is positive or hypoglycemia occurs independently of food intake. Subsequently, the criteria of Anlauf et al., Raffel et al., and Klöppel et al. should be applied to establish a histopathological diagnosis. According to our discussion above, we are of the opinion that “islet dysplasia” or “islet cell atypia” in a general sense, i.e., meaning an abnormal histologic appearance of the islets (and not necessarily a neoplastic precursor), would best describe the morphological findings. This would abolish the ill-defined term “nesidioblastosis” and avoid the reporting of inconsistent findings such as “islet cell hyperplasia”.

## 4. Nesidioblastosis and Islet Cell Hyperplasia in Other Adult Diseases

Not all patients diagnosed with nesidioblastosis or islet cell hyperplasia of the pancreas simultaneously exhibit symptoms of hyperinsulinemic hypoglycemia. This is both a consequence of adaptive changes in the endocrine pancreas in response to other diseases and of the inconsistent use of these denominations. In the more recent medical literature, the term nesidioblastosis is, however, more or less restricted to disorders related to endogenous hyperinsulinism (see above).

In infants, hypertrophy or hyperplasia of the islets of Langerhans was reported in association with many clinical conditions, including infants of diabetic mothers, Beckwith–Wiedemann syndrome, erythroblastosis fetalis, Zellweger spectrum disorder, α-Thalassemia, Donohue syndrome, Tyrosinemia, cyanotic congenital heart disease, long-term parenteral nutrition, MEN2 syndrome, Smith–Magenis syndrome and, of course, PHH/CHI/focal adenomatosis [334].

Adult patients with nesidioblastosis/islet cell hyperplasia can also exhibit a huge variety of comorbidities.

The relationship between insulinoma, nesidioblastosis/islet cell hyperplasia, and clinical symptoms of hypoglycemia in the adult population was established early. This is sometimes called “background nesidioblastosis” [231]. The first case was reported by Knight et al. in 1967 [75]. Our literature research identified at least 54 cases of insulinoma with coexisting, significant changes in the morphology in the remaining islets of Langerhans [16,40,58,68,71,75,109,121,130,170,177,182,215,218,231,266,276,283,285,286,316,318]. Two of the patients were found to have metastatic disease (a 40-year-old woman and a 34-year-old man [109,266]). The case presented by Campbell et al. in 1985 additionally showed serological evidence of autoantibodies to islet cells [285]. If these antibodies are related to the islet cell hyperplasia, duct-associated endocrine proliferation, or budding of islets remains unclear. A 58-year-old woman with insulinoma, chronic pancreatitis, villous adenomatosis of the pancreatic duct, and islet cell hyperplasia of A, B, and somatostatin-producing D cells was described in 1993 [318]. When applying the above-discussed strict definition of adult-onset nesidioblastosis, this case could not be classified as nesidioblastosis. A large populational study from Japan reported 1085 cases of organic hyperinsulinism in 1998, of whom 44 were diagnosed with nesidioblastosis. Sixty percent of these patients had simultaneous insulinoma [40]. Multiple insulinomas and nesidioblastosis are also a feature of MEN1 (see below). Dauriz and colleagues reported an especially interesting case of a 63-year-old woman who underwent distal pancreatectomy for insulinoma 4 years before the onset of recurrent hypoglycemia symptoms [231]. The patient experienced an insulinoma relapse due to background nesidioblastosis and was treated with a subtotal pancreatectomy since conservative treatment with diazoxide and pasireotide was not successful. This case illustrates a potential pathogenetic relationship between islet cell hyperplasia/nesidioblastosis and the emergence of insulinomas. It is still a matter of debate if, at least in some cases, the changes in the islets precede the formation of the actual tumor and if nesidioblastosis should therefore be regarded as a (facultative) pre-neoplasia (see below) [180,335].

In adults, findings of islet cell hyperplasia or nesidioblastosis were also reported independently of hyperinsulinism.

Glucagon is produced in the A/α/alpha cells of the islets of Langerhans. Tumors arising from these cells are called glucagonomas. Hyperglucagonemia can also arise from diffuse hyperplasia of the α-cells. A clinicopathological classification of related disorders accompanied by α-cell hyperplasia, as well as a collection of cases, was presented by Ouyang et al. in 2011 and Yu in 2014 [96,336]. We identified 18 cases of α-cell hyperplasia and/or glucagonoma associated with nesidioblastosis [38,74,88,96,115,141,162,163,164,179,189,213,298,312,336]. To the best of our knowledge, the first report of nesidioblastosis with scattered α-cells throughout the acinar tissue was presented by Balas et al. in 1988 [298]. A 35-year-old woman was diagnosed with a tumor in the head of the pancreas and was shown to have an additional silent glucagonoma as well as α-cell nesidioblastosis. Two cases of malignant glucagonoma with nesidioblastosis were reported in 1991. While one presented with β-cell hyperplasia, the other presented with α-cell hyperplasia [312] (the latter cited in [96]). Furthermore, two cases described by Brown et al. became clinically apparent with diabetes mellitus, as would be expected from an excess of glucagon [38]. A necrolytic migratory erythema was also reported in association with diffuse α-cell hyperplasia, which was diagnosed as “diffuse glucagonoma” since no actual tumor mass was found [88]. An unusual case of hyperinsulinemic hypoglycemia was reported by Roberts et al. in 2012 [164], where a 56-year-old male presented with glucagonoma and a glucagon-like peptide 1 (GLP-1) secreting metastatic mass (“GLPoma”). First, the patient presented with diabetes (most likely due to the predominance of the glucagonoma) and later developed hyperinsulinemic hypoglycemia, which is probably the consequence of the GLP-1 excess, which acts as a potent insulin secretagogue. A coexistence of α-cell hyperplasia with renal cell carcinoma and a cystic lesion of the pancreas in a 56-year-old man was also described [179]. Another notable case was a 35-year-old woman who suffered from recurrent acute pancreatitis and glucagonoma with α-cell nesidioblastosis [189]. A middle-aged woman was diagnosed with a microadenoma showing glucagon expression. Of interest, this patient reported by Schwetz et al. was diagnosed with hyperinsulinemic hypoglycemia and required distal pancreatectomy and pasireotide postoperatively [213]. The cases of α-cell nesidioblastosis and/or glucagonoma thus show various clinical presentations ranging from diabetes mellitus to severe hypoglycemia. As proposed by Yu, these diseases can be classified as reactive, functional, and non-functional. Accordingly, reactive α-cell hyperplasia arises from defects in glucagon receptor signaling and is associated with nonspecific symptoms such as abdominal pain. The pancreatic mass is often an incidental finding, as even extreme hyperglucagonemia might not be associated with a clinical glucagonoma syndrome. If the mutation (e.g., P86S homozygous inactivating mutation of the human glucagon receptor) is clearly defined, this clinical picture is also known as Mahvash disease [337]. Functional glucagonoma syndromes show a characteristic clinical appearance with elevated glucagon levels but exhibit no gross pancreatic neuroendocrine tumor (PNET). Nonfunctional cases have no specific symptoms, do not have elevated glucagon levels, have no glucagonoma syndrome, and might or might not have a gross PNET. Mutations in the latter cases are not known [336]. From the 12 cases reported by Yu (all included in our summary), 5 were classified as reactive, 1–2 as functional, 3 as nonfunctional, and 2 could not be classified.

An association of islet cell hyperplasia without symptoms of hypoglycemia was reported in a 41-year-old woman suffering from von Hippel–Lindau disease [83]. The patient also had bilateral pheochromocytoma, renal cell carcinoma, and multiple lesions in the pancreas (including an endocrine carcinoma).

Cases of MEN1 with concomitant pancreatic nesidioblastosis or islet cell hyperplasia have been repeatedly reported (according to our literature research criteria described above, we found a total of 28 patients [20,28,42,80,172,245,277,288,294]). In some of them, coexisting insulinomas were found, as in the two cases reported by Proye et al. [80]. However, not all patients with MEN1 and islet cell changes exhibited clinical symptoms of hypoglycemia. The patient reported by Franksson et al. in 1960 is presumably the first patient with MEN1 reported to have islet cell hyperplasia. Additionally, chief-cell hyperplasia of the parathyroid, adenomata of the pancreas, and gastro-duodenal ulcers were found [42]. It is likely that this case is actually an overlap syndrome of MEN1 and Zollinger–Ellison syndrome (MEN1 with gastrinoma) [338]. Members from a MEN1-family described in 1972 presented with excessive insulin secretion, and the individuals who underwent pancreatectomy also had islet cell hyperplasia [172]. A 30-year-old man with MEN1, who was also diagnosed with nesidioblastosis and hypoglycemia, had an additional growth hormone-releasing hormone (GHRH) producing tumor, hyperparathyroidism, hyperprolactinemia, and multiple endocrine pancreatic tumors [294]. A suspected MEN1/MEN2 overlap syndrome became clinically apparent through a medullary thyroid carcinoma, watery diarrhea, and flushing. Pancreatic nesidioblastosis of the pancreatic polypeptide (PP) expressing cells were recognized (see below) [292].

Nesidioblastosis or islet cell hyperplasia in association with Zollinger–Ellison syndrome is also a relatively common finding (according to our literature research criteria described above, we identified a total of 64 patients with (Pseudo) Zollinger–Ellison syndrome [32,42,64,86,107,139,183,194,262,271,284,303,339]). The study by Ellison et al. is especially interesting since a large number of patients was studied, of whom 10% showed islet cell hyperplasia [64]. In most cases, pancreatic islet hyperplasia was associated with a morphologically detectable gastro-pancreaticoduodenal gastrinoma (e.g., Larsson et al. 1973 [183]). Of interest, increased β-cell replication was reported in islets directly adjacent to the gastrinoma but not in islets further away from the tumor [339]. This suggests that there might be a paracrine, a secretory factor that contributes to the morphological changes seen in those pancreata. Above, we could identify a possible case of Pseudo Zollinger–Ellison syndrome with islet cell hyperplasia [262]. This means that there is no macroscopic or microscopic tumor detectable, but hypergastrinemia arises from hyperplasia of gastrin-producing cells. The case described by Varas Lorenzo et al. might be an example of hypergastrinemia related to islet cell hyperplasia causing a Pseudo Zollinger–Ellison syndrome.

Verner–Morrison syndrome is defined by secretory diarrhea, hypokalemia, and hypochlorhydria. Islet cell hyperplasia has been reported in some cases (according to our literature research criteria described above, we identified a total of 19 patients with definite or suspected (Pseudo) Verner–Morrison syndrome/VIPoma [44,118,138,161,205,258,261,264,282,310]). While Sircues et al. described an overrepresentation of β-cells in their patients, Jacobs et al. reported non-β-islet hyperplasia [118,161]. Verner et al. agree with the finding by Jacobs and colleagues [205]. As Verner–Morrison syndrome is mostly caused by tumors, which induce an overproduction of vasoactive intestinal peptide (VIP), also called VIPomas, the report of increased VIP blood levels with concomitant islet cell hyperplasia by Schwarz et al. was important for establishing the causal relationship [258]. Tomita et al. were the first to report that the cell population responsible for the observed islet hyperplasia in clinically suspected Verner–Morrison syndrome are, in some instances, the PP-producing cells [264]. They proposed that, in the absence of a VIPoma, PP excess due to PP-cell hyperplasia can cause a similar clinical picture, which is consequently termed Pseudo Verner–Morrison syndrome. PP-cell hyperplasia in association with A-cell hyperplasia was later reported by the same group [282]. A concise review of A- and PP-cell hyperplasia of the pancreas and their clinical implications were presented by Ouyang and colleagues in 2011 [96]. From a diagnostic point of view, the finding by Francesconi et al. is interesting [138]. A 60-year-old man presented with fecal urgency and diarrhea and was found to have a focal pancreatic uptake in ^111^In-Pentetreotide scintigraphy. Clinically, a VIPoma was suspected. The lesion was found to be a (focal?) nesidioblastosis without any signs of hyperinsulinism. The symptoms persisted after surgery, and chromogranin A levels remained within the normal range. Although clinically highly suggestive of Verner–Morrison syndrome, no neuroendocrine tumor was found, and the nesidioblastosis seemed to be unrelated to the symptoms. Careful differential diagnosis, therefore, remains highly important. Additionally, PP cell hyperplasia without diarrhea or suspected VIPoma was reported in two elderly males [100,113]. One of them presented with pseudo-obstruction of bowels and had additional microadenomas of the pancreas. None of the patients had symptoms of hypoglycemia.

In areas of pancreatic fibrosis and during chronic pancreatitis, islet cell hyperplasia is commonly found and is most likely a reactive adaptation to the chronic inflammatory stimuli and a sign of regenerative processes [17,96,129,291,328]. Pancreatic duct obstruction/ligation was shown both experimentally and clinically to induce nesidioblastosis [291,340,341,342]. In rare instances, pancreatitis/pancreatic fibrosis was associated with hyperinsulinemic hypoglycemia. Such a case of a 20-year-old man suffering from recurrent abdominal pain, vomiting, chronic familial pancreatitis, and hyperinsulinemic hypoglycemia with histologically proven nesidioblastosis was reported by Wig et al. in 2008 [129]. In another case of a 67-year-old man, calcified pancreatitis was associated with hyperparathyroidism and hyperplasia of α-cells of the pancreas (Paloyan et al., 1967; cited in [96]).

Morphological studies with pancreata from cystic fibrosis patients have been conducted by different authors [322,343,344]. Nesidioblastosis, in this case, meaning the neo-formation of islets, might be a compensatory mechanism in this disease and protract the onset of diabetes mellitus. Brown et al. described patterns of nesidioblastosis in cystic fibrosis patients in 1971 (cited in [128]).

An association of intraductal papillary mucinous neoplasm (IPMN) or pseudopapillary neoplasm of the pancreas has been reported at least four times [145,158,220,345]. Two of these cases had additional neuroendocrine tumors, and one exhibited α-cell hyperplasia [220,345].

Islet cell hyperplasia/nesidioblastosis is also reported in response to drug intake. An early post-mortem study by Bloodworth conducted in 1963 describes the neo-formation of islets, islet hyperplasia, an increase in α-cells, and a decrease in β-cells of adults treated with the sulfonylurea compound tolbutamide [53]. If these patients had any symptoms related to hypoglycemia, it was not clear. In 1984, a case of a 25-year-old woman with hyperinsulinemic hypoglycemia with pathohistological signs of islet cell hyperplasia and nesidioblastosis after subtotal pancreatectomy was also reported in response to chlorpropamide treatment (sulfonylurea). Moreover, three cases of narcotic addicts suffering from nesidioblastosis are documented [97,125]. All three women presented with hyperinsulinemic hypoglycemia, and two were HIV positive but without retroviral therapy.

The association of nesidioblastosis or islet cell hyperplasia with pancreatitis, pancreatic fibrosis, cystic fibrosis, the tumor syndromes MEN1, and von Hippel–Lindau syndrome, as well as with Zollinger–Ellison syndrome, Verner–Morrison syndrome, glucagonoma, insulinoma, and sulfonylurea intake are well-established and, although not experimentally proven, thought to be related to the pathophysiological bases of the diseases. The associations between nesidioblastosis or islet cell hyperplasia and other diseases, which are described next, are mostly based on single/a few reports and might, therefore, rather present simple comorbidities that are not related causally. For reasons of completeness, they are, however, presented here.

An autopsy evaluation of the pancreas of a 54-year-old man who suffered from multiple malignancies and who was a kidney transplant recipient revealed islet cell hyperplasia [252]. Clinical symptoms were, however, not reported. Yeh et al. presented the case of an autopsied 72-year-old woman with myelodysplastic syndrome and nodular glomerulosclerosis, who was, however, never diagnosed with diabetes [49]. She had a history of symptomatic hyperinsulinemic hypoglycemia, and the pancreas showed signs of nesidioblastosis/islet cell hyperplasia. It is plausible to assume that hyperinsulinism might have reversed a preexisting type II diabetes. A 36-year-old man with end-stage chronic kidney disease presented with hyperinsulinemic hypoglycemia [230]. Since insulin clearance is impaired in chronic kidney disease, SACS was performed to observe whether there was an inadequate insulin response to calcium stimulation. The test was positive and subtotal pancreatectomy was performed, revealing islet cell hyperplasia/nesidioblastosis. A 73-year-old man, who depended on dialysis for 18 years, also suffered from hyperinsulinemic hypoglycemia [244]. Duodenopancreatectomy was not sufficient for symptom control. The patient is now treated with continuous subcutaneous octreotide infusion and corticosteroids. The histopathological evaluation confirmed adult-onset nesidioblastosis. These four cases are all related to kidney diseases, but we are not aware of any common pathophysiological mechanism.

Larsson et al. reported a case of pernicious anemia and atrophic gastritis with concomitant nesidioblastosis of the pancreas [253]. Chronic laxative abuse was also shown to be associated with an increased number of islets and prominent ductulo–insular complexes in a 39-year-old man [255]. A 59-year-old woman with hyperinsulinism, hypoglycemia, and hyperglucagonemia was shown to have islet hyperplasia [267]. She additionally suffered from biliary cirrhosis and had a portocaval anastomosis and a gastrojejunostomy. This case of islet cell hyperplasia might either be related to some undetected neuroendocrine neoplasia or represent an early case of gastric surgery-associated nesidioblastosis (see below).

Autoimmune phenomena in the context of nesidioblastosis were reported in at least three instances, including one of the above-discussed cases with coexisting insulinoma [195,274,285]. One patient with a history of scleroderma suffered from anti-insulin receptor antibody-induced diabetes mellitus [274]. Islet cell hyperplasia was detected in the pancreas, and the authors concluded that the neo-formation of endocrine cells might represent a compensatory reaction to antibody-induced diabetes. A woman described by Woo et al. exhibited simultaneous nesidioblastosis and insulin autoantibodies [195]. Clinically, she presented with hyperinsulinemic hypoglycemia. Such rare instances might represent overlap syndromes of Hirata´s disease and adult-onset nesidioblastosis, as the insulin–autoantibody syndrome can provoke both episodes of hypoglycemia and hyperglycemia. If the latter phenomenon dominates, islet hyperplasia could indeed be a compensatory mechanism for developing hyperglycemia. Alternatively, the antibodies could exert a stimulatory function on the islet cells and thereby favor proliferation or hypertrophy. The exact mechanism, however, remains to be determined.

Five adults with genetic forms of α1-antitrypsin deficiency were shown to have islet cell hyperplasia and/or nesidioblastosis [289]. Symptoms of hypoglycemia were not reported.

Nesidioblastosis in heterotopic pancreata was found twice [110,239]. A 24-year-old woman had an enteric intussusception due to a jejunal heterotopic pancreas with nesidioblastosis but no hypoglycemia [110]. A 32-year-old man had pancreatic nesidioblastosis and heterotopic pancreatic tissue with nesidioblastosis, accompanied by hyperinsulinemic hypoglycemia [239].

Of interest, nesidioblastosis has been reported as coexisting disease in diabetic patients. Thus far, 13 cases have been described in the literature [17,27,57,65,66,67,75,101,132,180,233]. In some of the patients, the nesidioblastosis-related hyperinsulinemia led to a reversal of the diabetic syndrome, and insulin therapy could be discontinued. The others still needed diabetic treatment.

Nesidioblastosis in neuroendocrine tumors other than insulinoma, glucagonoma, and gastrinoma has been described [197,200,229,236]. A 59-year-old woman was diagnosed with a non-functioning PNET and hyperinsulinemic hypoglycemia due to islet cell hyperplasia [197]. Although D-cell hyperplasia has probably been only reported once [318], a case of metastasizing somatostatinoma has been described by Wiesli et al. [229]. Although the patient had episodes of hyperglycemia (somatostatin can inhibit both glucagon and insulin secretion), postprandial hyperinsulinemic hypoglycemia was also detected and related to postmortem findings of islet cell hyperplasia. This is interesting since it indicates different functions of somatostatin during different nutritional phases.

Single case reports documenting islet cell hyperplasia/nesidioblastosis with the following diseases can also be found in the medical literature: Choristoma (no reported hyperinsulinism) [304]; fibrocystic pancreatic atrophy with concomitant pancreatic carcinoma (no reported hyperinsulinism) [321]; familial adenomatous polyposis (FAP, with hyperinsulinemic hypoglycemia) [66]; after pancreas transplantation for diabetes mellitus type I (with hyperinsulinemic hypoglycemia) [67]; orbital lymphoma, hypopituitarism, and secondary adrenal insufficiency (with hyperinsulinemic hypoglycemia) [70]; Sheehan syndrome (with hyperinsulinemic hypoglycemia) [198]; Hashimoto thyroiditis with chronic renal failure, incidental adrenal mass, and ovarian thecal metaplasia (no reported hyperinsulinism) [76]; and a patient with short bowel syndrome with GLP1-agonist treatment for diabetes mellitus type II (with hyperinsulinemic hypoglycemia) [233].

As can be seen from this extensive discussion, there is a huge spectrum of diseases associated with nesidioblastosis/islet cell hyperplasia. While some findings have been reported repeatedly and might thus be connected pathophysiologically, other coexistences might just be incidental comorbidities. Since there are cases of A, B, and PP cell hyperplasia with different clinical symptoms, it can be concluded that these represent different clinical and pathophysiological entities, albeit with some shared histopathological characteristics. We, however, think that the nomenclature should be adjusted accordingly and that histopathological findings should be more strictly correlated with the clinical picture to avoid inaccurate reporting as “nesidioblastosis” or “islet cell hyperplasia”.

## 5. Etiology and Pathophysiology of NIPHS/Nesidioblastosis with Hyperinsulinism

The etiology of NIPHS/nesidioblastosis with hyperinsulinism in the adult is largely unknown. For PHH/CHI, defined disease-causing mutations in various genes are known [346]. To date, ten genes have been identified that are involved in different, non-syndromic clinical forms of the disease: *ABCC8* (ATP binding cassette subfamily C member 8; modulates ATP sensitive potassium channels and therefore interacts with KCNJ11), *KCNJ11* (ATP-sensitive potassium channel = K_ir_6.2/K_ATP_; interacts with the sulfonylurea receptor), *GCK* (Glucokinase), *HK* (hexokinase), *GLUD-1* (Glutamate Dehydrogenase type 1), *HADH1* (Hydroxyacyl-coenzyme A Dehydrogenase = *SCHAD* = short-chain-3-hydroxyacyl-coenzyme A Dehydrogenase), *SLC16A1* (solute carrier family 16, member 1 = *MCP1* = monocarboxylate transporter 1), *HNF1A* (hepatic nuclear factor 1A), *HNF4A* (hepatic nuclear factor 4A), and *UCP2* (uncoupling protein 2). Due to different pathophysiological mechanisms, the mutations in these genes also determine the patients´ responses to specific treatments such as diazoxide administration. A concise discussion of the genetics of CHI and CHI associated with other syndromic diseases is provided by Rosenfeld et al. and by Gilis-Januszewska et al. [347,348].

An important question that arises when discussing the differences between CHI/PHH and adult-onset nesidioblastosis is, which age of symptom onset can be regarded as an adult? The case presented in our recent Case Report (see Dieterle et al., 2023; submitted to *Biomedicines*) was clinically apparent for the first time at the age of 15. In this context, a study by Dahms et al. from 1980 contributes important information [263]. They analyzed cases of hyperinsulinemic hypoglycemia with age-matched autopsy controls. A certain degree of “nesidioblastosis” (in this case, meaning the neo-formation of islets and their association with the exocrine ducts) was found to be normal in newborns and early infancy. Quantitatively, this feature significantly decreases until the age of approximately 3 years. CHI/PHH usually becomes clinically apparent within the first days, weeks, or months of life [331]. According to the data obtained from the literature, we, therefore, think that new-onset hyperinsulinemic hypoglycemia above the age of 3 years without one of the characteristic mutations might be seen as a form of late/”adult”-onset hyperinsulinism. This should be regarded as pathophysiologically different from the genetic forms described above. Late-onset NIPHS/nesidioblastosis/islet hyperplasia/islet dysplasia might therefore be a better characterization than adult-onset and would thus include older children, adolescents, and adults. It would also eliminate the uncertainty of the exact onset of symptoms as in our presented case, where the patient was an adult at diagnosis but not at the expected beginning of the disease. A recent article by Castillo-López and colleagues also discussed this issue. They reported a 15-year-old male with hyperinsulinemic hypoglycemia and histological findings typical of nesidioblastosis [247]. This case was classified as “adolescent” according to their definition (children: aged <15 years; adolescents: aged 15–21 years; adults: aged >21 years). The authors performed a literature research aiming at the adolescent population of nesidioblastosis patients and found 41 cases, 24 of which were not retrieved by our search strategy but would be classified as adult when using our definition [349,350,351,352,353,354,355,356]. This again underscores the problem associated with non-standardized definitions of disease entities and the overlap of pediatric, adolescent, and adult patient collectives.

In older children, adolescents, or adults, sporadic or idiopathic cases that are not associated with a genetically defined disease, such as MEN1 or bariatric surgery, are not well understood [12]. Idiopathic cases with *MENIN* mutations, the gene related to MEN1, were never found [11]. Mutations in subunits of the sulfonylurea receptor (*SUR*) were also not described in the literature [45]. The same is true for *MODY2/3* (maturity-onset diabetes of the young type 2 and 3) genes [101]. A definite genetic cause of adult-onset hyperinsulinemic hypoglycemia with islet cell hyperplasia/nesidioblastosis has only been found in a limited number of adults or cases with symptom onset at school age [187,212]. All the respective mutations were described as activating glucokinase (*GCK*) variants. To the best of our knowledge, the first of these cases was published in 1998 by Glaser and colleagues, who described a p.Val455Met mutation in a family presenting with hyperinsulinemic hypoglycemia (listed as rs104894012 in the NCBI SNP database) [357]. Euglycemia was achieved via diazoxide treatment in all patients. Christesen et al. reported a second activating *GCK* variant, namely p.Ala456Val, which also affected a 42-year-old woman (listed as rs104894014 in the NCBI SNP database) [358]. Due to a lack of clinical symptoms (the only finding was fasting hypoglycemia, classified as “relative hyperinsulinemia”), the patient never needed treatment. Activating mutations of *GCK* in adults were also found at positions p.Thr65Ile and p.Trp99Arg, as reported in [359]. Only the woman reported in this study needed treatment with pancreatic head resection and diazoxide. Five adult cases (all from one family) were shown to have activating p.Val389Leu mutations (listed as rs1350717554 in the NCBI SNP database) [187]. All patients suffered from hypoglycemia, but only three were symptomatic. Histopathological evaluation of the pancreata was, however, not available since the patients were treated with diazoxide. Lu et al. found two patients with activating GCK mutations who had hyperinsulinemic hypoglycemia at school age. These cases illustrate that simple age-dependent clinical evaluation of patients is sometimes unsuccessful in properly distinguishing adult/late-onset hyperinsulinemic hypoglycemia/NIPHS from inherited forms. Therefore, thorough genetic work-up, especially in patients presenting at a relatively young age but not directly in the newborn/early infancy period, should always be considered. An intriguing case of an obese (BMI 49.1 kg/m^2^) 22-year-old was recently reported by Koneshamoorthy and co-workers [248]. The patient presented with hyperinsulinemic hypoglycemia and received a distal pancreatectomy, which, however, did not lead to symptom relief. A review of the medical history revealed that neonatal hypoglycemia was diagnosed in this patient (macrosomic newborn) and treated with diazoxide until the age of 3. Genetic testing revealed a novel activating *GCK* variant (c.269A>C; p.Lys90Thr), which was also found in the mother (partial pancreatectomy at age 6 for hypoglycemic seizes), his sister (hyperinsulinemic hypoglycemia discovered upon monitoring for gestational diabetes), and the nephew (neonatal hypoglycemia treated with diazoxide). A postoperative treatment attempt with diazoxide was unsuccessful, while pasireotide proved suitable to control hypoglycemia. Functional studies of islet cells from this patient are reported below. This case underscores the clinical heterogeneity (asymptomatic cases are possible; the degree of symptoms might vary with hepatic regulation of GCK) and different ages of onset of *GCK*-related nesidioblastosis, which needs to be considered when evaluating patients suffering from hyperinsulinemic hypoglycemia at any age.

**Figure 1:**
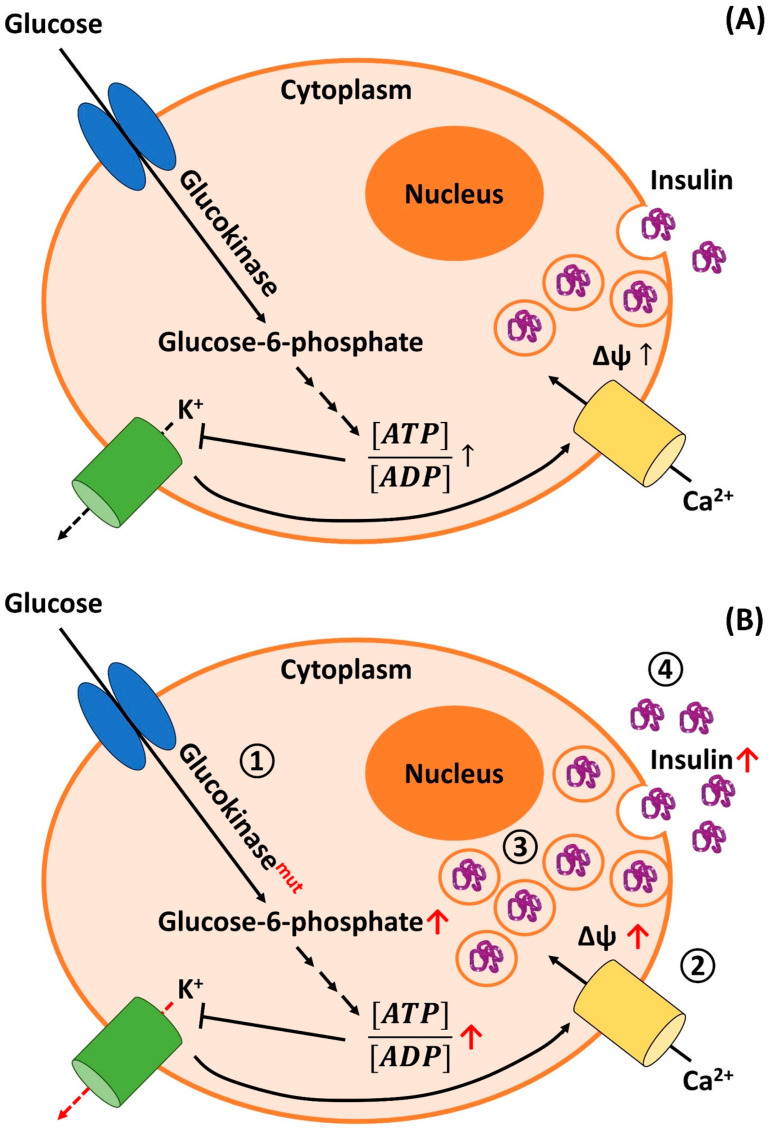
Physiological mechanism of insulin secretion in human pancreatic β-cells (A) and pathophysiological aspects of adult-onset nesidioblastosis/NIPHS: (**A**) Glucose enters the pancreatic β-cells through glucose transporting proteins (GLUT) subtypes 1 and 3 [391]. Glucose is phosphorylated via Glucokinase (GCK; also termed hexokinase IV), leading to the production of Glucose-6-phosphate, which is trapped intracellularly. Subsequent chemical reactions (small arrows) are part of the glycolysis, the citric acid/Krebs cycle, and the respiratory chain (oxidative phosphorylation). Finally, complete oxidative glucose metabolism leads to the production of adenosine triphosphate (ATP). A relative increase in intracellular ATP concentrations upon glucose intake leads to a rise in the ATP:ADP (adenosine diphosphate) ratio. ATP inhibits the activity of the K_ir_6.2/K_ATP_ channel (green barrel). A reduced efflux of potassium (K^+^) ions results in a rise in the membrane potential (Δψ). When reaching the threshold potential, voltage-gated calcium (Ca^2+^) channels (yellow barrel) open. The influx of calcium stimulates the fusion of preformed insulin-containing vesicles with the plasma membrane, i.e., insulin release is triggered. (**B**) In patients suffering from adult-onset nesidioblastosis/NIPHS, different pathophysiological mechanisms are discussed (also see main text; main metabolic changes as well as mutated enzymes are indicated in red). (1) Activating mutations of the *GCK* (Glucokinase^mut^) gene lead to a higher activity of the corresponding enzyme. This increases the metabolic flux through the oxidative metabolic pathways, which raises the ATP:ADP ratio. This, in turn, leads to an ATP-dependent inhibition of K^+^ channels, which reduces the potassium efflux from the cell (red dashed arrow). (2) β-cells from adult-onset nesidioblastosis/NIPHS patients were shown to have a higher resting membrane potential Δψ. This results in a higher open-state probability of voltage-gated calcium channels. (3) β-cells from patients may possess an overall higher amount of intracellular insulin synthesis and intracellularly stored insulin. (4) A higher basal insulin secretion rate was also reported for adult-onset nesidioblastosis/NIPHS cells. To summarize, all mechanisms (1)–(4) increase insulin secretion from the islets of Langerhans, resulting in hyperinsulinemic hypoglycemia and the associated clinical symptoms.

Although not exclusively studied in the context of adult-onset nesidioblastosis/NIPHS, the molecular mechanism of hyperinsulinism related to *GCK* mutations might be similar for pediatric CHI/PHH and adult NIPHS cases. Structurally, some of the known *GCK* mutations were shown to map to the allosteric activator site of the enzyme. Mechanistically, there are two groups of activating *GCK* mutations leading to hyperinsulinemic hypoglycemia: one group leads to a “left shift” of glucose binding, i.e., an increased glucose affinity of the enzyme (e.g., p.Thr65Ile and p.Ala456Val). The other group, e.g., p.Val455Met and p.Trp99Arg, favor the isomerization of the enzyme into its active form [360,361]. The common final path of both mutation groups is the same: the glucose metabolizing activity of the pancreatic β-cells increases, resulting in a rise in the glycolytic and citric acid cycle/respiratory chain activity, which is accompanied by an increase in the concentration ratio of ATP and ADP ($\frac{\left[ATP\right]}{\left[ADP\right]}↑)$. ATP inhibits the K_ir_6.2/K_ATP_ activity, which depolarizes the cells and enhances the open-state probability of voltage-gated calcium channels. Calcium influx enables the fusion of preformed insulin-containing vesicles with the plasma membrane. In summary, the glucose-stimulated insulin secretion of the β-cells increases, giving rise to hyperinsulinemic hypoglycemia (see Figure 1) [347,362].

Apart from genetics, environmental causes have been proposed as triggers for late-onset nesidioblastosis [12]. However, the impact of, e.g., nutritional factors such as carbohydrate intake or exposure to nutritional or environmental toxins has never been investigated systematically.

Experimental findings on late-onset nesidioblastosis are scarce. Campbell and colleagues were able to establish cell cultures of islet cells from a patient with islet hyperplasia. Of interest, the islets could be grown in culture for up to 60 days, whereas normal islet cells usually die after 14 days. Overgrowth of the islets was also reported in the culture system [285]. Similar experiments were performed by Roncari and co-workers. They observed faster growth of adult hyperplasia-derived islet cells in culture in comparison with control conditions [296]. Functionally, the in vitro studies also confirmed that nesidioblastosis-derived islet cells have a higher basal insulin secretion, an overall higher insulin content, and express more insulin mRNA [37]. The insulin secretion persisted throughout the culture period of an infant-derived nesidioblastosis cell line, which is not the case upon a long-term passage in normal islet cells [363]. Insulin secretion and peripherally detectable C-peptide levels in comparison with insulinoma are nonetheless lower [112]. Islet neogenesis-associated protein (INGAP) expression in some adult-onset nesidioblastosis patients was increased, which points in the direction of increased neo-formation of endocrine cells in the pancreas. However, the key pancreatic transcription factors NK6 homeobox 1(Nkx6.1) and pancreatic and duodenal homeobox 1 (PDX1), as well as GLP-1, showed no abnormalities in nesidioblastosis patients [91,106]. The expression pattern of several growth factors and their receptors was also studied in nesidioblastosis. An increase in insulin-like growth factor 2 (IGF2), IGF 1 receptor subtype alpha (IGF1Rα), and transforming growth factor β receptor 3 (TGFβR3) expression was detected, whereas no difference was apparent in epidermal growth factor receptor (EGFR), TGFβ1, or TGFβ2 levels [137]. Of interest, the IGF2 overexpression was only present in idiopathic nesidioblastosis but not in post-bariatric surgery cases. In the above-discussed case by Koneshamoorthy and co-workers, single-cell transcriptomic studies uncovered an elevated insulin (*INS)* and creatine kinase B (*CKB*) expression, whereas protein delta homolog 1 (*DLK1*) and neuropeptide Y (*NPY*) expression was downregulated [248]. DLK1 and NPY inhibit the switch of islet cells from proliferation to differentiation. Thus, their downregulation indicates that the mutation, in this case, does not lead to a hyperproliferation or genuine neoplasia of islet cells. Genes associated with cell cycle progression, β-cell differentiation, or glycolysis did not differ between patient-derived cells and control conditions. The same was true for the insulin receptor, insulin receptor substrate 1 (*IRS1*), insulin receptor substrate 2 (*IRS2*), and *KIAA1324*.

Electrophysiological experiments were also conducted with nesidioblastosis-derived cells [149]. Even at low glucose concentrations (3 mmol/L), depolarization, as well as action potentials, were registered at the β-cell membrane. While the resting membrane potential at 3 mmol/L in normal islet cells is around −60 to −70 mV, nesidioblastosis-derived cells were depolarized to −30 to −5 mV. This is unusual since low glucose values normally suppress endogenous insulin secretion. The function of the K_ir_6.2 channel was, however, inconspicuous. These observations, although only reported in a single study, propose a molecular mechanism for the clinically observed functional β-cell disorder that not necessarily depends on an increase in β-cell mass and makes the diagnosis of nesidioblastosis independent of actual islet cell hyperplasia. The cells of the patient from the Koneshamoorthy study were also subjected to electrophysiological analyses [248]. Cytoplasmic Ca^2+^ concentrations of nesidioblastosis-derived and control cells were not significantly different at baseline. However, upon glucose administration, there was a remarkable increase in the calcium response in nesidioblastosis-derived cells, which consecutively led to higher insulin secretion. Although this case cannot be classified as genuinely adult, it might nonetheless contribute to a pathophysiological understanding of the disease.

Since the nuclei of the β-cells in nesidioblastosis were shown to be enlarged, which is one major histopathological diagnostic criterion, some authors assumed that the DNA content of these islet cells might be increased [333]. Flow cytometric testing of this hypothesis revealed that, in contrast to β-cell derived adenomas and carcinomas, which are often aneuploid, nesidioblastosis-derived cells are diploid [23].

Concerning the formal pathogenesis, a hyperplasia–dysplasia–adenoma (carcinoma) sequence was also proposed in the context of islet cell hyperplasia [335]. According to this model, an external or internal stimulus would induce the proliferation and/or hypertrophy of islet cells. Genetic and morphological changes in the respective cells would represent the next step, called dysplasia. A true adenoma, i.e., a neoplastic entity, would arise subsequently. Malignant transformation of an insulinoma would finally lead to metastatic, cancerous disease. This model could explain the frequently observed coexistence of islet hyperplasia and insulinoma. However, the opposite, that insulinomas lead to a paracrine stimulation of islet growth and/or proliferation of the islets of Langerhans, is also possible. Hyperplasia of ghrelin and obestatin immunoreactive cells at the periphery of islets has been reported and might contribute to such phenomena [186]. Both possibilities have never been adequately challenged experimentally [316]. There is only one study reporting the progression from nesidioblastosis/islet cell hyperplasia to a pancreatic tumor in SV40 large T transgenic mice [364]. The significance of this experimental finding to the human disease is, however, questionable since SV40 large T exhibits transforming activity in many cell types.

Other authors suggested that a lack of somatostatin, which inhibits β-cell function, leads to an unrestrained proliferation of islets. As discussed in the context of autoimmune phenomena and nesidioblastosis, circulating autoantibodies against islet cell epitopes could also transmit pro-proliferative stimuli and lead to hyperplasia [316].

The functional outcome of the β-cell disorder has also been intensively discussed in the literature [33]. Since a definitive increase in β-cell mass has not been convincingly shown, a quantitative increase in the number of islet cells is most likely not the cause of hypoglycemia. A relative increase in β-cells, i.e., an overrepresentation in comparison with A, D, and PP cells, also does not seem to be the main mechanism of hyperinsulinemic hypoglycemia since many authors did not find such a shift [11]. Changes in islet architecture, which could be a consequence of or result in aberrant patterns of paracrine signaling, could, however, contribute to inadequate insulin secretion. Additional changes in functional regulation, whose morphological correlate might be the changes in the β-cell nuclei, have also been proposed.

Of interest, nesidioblastosis with or without hyperinsulinemic hypoglycemia was reported in animals. Cases of a 2-day-old Simmental calf (no hyperinsulinism) [365], young Beagle dogs (no hyperinsulinism) [366], a 6-year-old castrated male Australian Shepherd (hyperinsulinemic hypoglycemia) [367], old dogs (some with hypoglycemia and/or hyperinsulinism) [368], two squirrel monkeys (hyperglycemia with glucagon-reactive nesidioblastosis) [369], a cat (hyperinsulinemic hypoglycemia) [370], and several aged horses (no documented hyperinsulinism) [371] were found in the literature. If the pathophysiology and etiology of these morphological findings are similar to the ones in humans remains to be established.

Animal models (e.g., hamster, mouse, monkey) or distinct cell culture models of nesidioblastosis/islet cell hyperplasia/proliferation have mainly been developed in the context of regenerative treatments for diabetes mellitus, experimental pancreatitis, or in the study of pancreatic malignancies [340,341,342,364,372,373,374,375,376,377,378,379,380,381,382,383,384,385,386,387]. Due to a lack of significant contribution to the understanding of the pathophysiology of human nesidioblastosis, this is not further discussed here.

Although not the direct topic of this review, some important findings concerning nesidioblastosis/islet cell hyperplasia after gastrointestinal surgery are discussed. For post-gastric bypass hyperinsulinemic hypoglycemia (PGBHH), the etiology and pathophysiology of hyperinsulinemic hypoglycemia seem to be different from idiopathic cases. The reports by Patti et al. and Service et al. in 2005 are generally thought to be the first cases of PGBHH. A prominent feature of the disease is postprandial hyperinsulinemic hypoglycemia [7,84]. However, we suppose that the case by Brennan et al. from 1980 might also be related to a gastroenterostomy [267]. Most patients reported with this syndrome underwent Roux-en-Y bypass surgery. According to our literature research, there are just single cases after vertical banded gastroplasty [84], duodenal switch [143], fundoplication [156], esophagectomy [127], or sleeve gastrectomy [234]. The existence of PGBHH with pancreatic nesidioblastosis is, however, under debate. Some authors deny its overall existence, whereas histopathological findings were consistent with the above-discussed criteria for adult-onset nesidioblastosis in many cases. Sometimes, the diagnosis is also established only clinically. An overlap with other (functional) hyperinsulinemic hypoglycemia syndromes after gastric bypass surgery, such as late dumping syndrome, is also likely. Supplementary Table S1 mostly contains cases with histopathologically confirmed nesidioblastosis/islet cell hyperplasia or a highly suggestive clinical presentation. We counted a total of 201 cases plus additional 135 cases discussed at a conference in Harvard [140] (it is not known if some of them were reported before or overlap otherwise) [5,7,84,99,102,103,105,114,114,116,119,120,122,124,127,133,137,140,142,143,144,145,153,154,155,156,157,165,167,168,169,171,174,177,188,190,191,193,201,204,207,209,217,223,224,234,235,237,243,267]. Where demographic data were available, a female:male ratio of approximately 4:1 was calculated, which might represent the fact that more women undergo bariatric surgery for morbid obesity or might be related to sex hormone-dependent mechanisms (see below) [388]. The post-bariatric population thus represents a considerable fraction of the patients treated for non-insulinoma hyperinsulinemic hypoglycemia in the adult population.

One characteristic of PGBHH is that it occurs with a delay of months to years after the surgical intervention (late dumping syndrome is mostly recognized much earlier). Generally, hypoglycemic symptoms after this type of surgery were reported to occur in about 2.6% postoperatively, with a high spontaneous recovery rate [389]. Patients after gastric bypass show elevated plasma levels of GLP-1 since nutrients have rapid contact with the distal ileum, where the GLP-1-producing L cells reside [84]. As GLP-1 induces β-cell proliferation in rodents, it was suspected that this mechanism might contribute to the observed syndrome. A concomitant decrease in plasma ghrelin, which is an inhibitor of pancreatic insulin secretion, was also recognized [390]. According to Meier et al., histological studies of pancreata from PGBHH patients did not reveal an increase in the β-cell area or neogenesis of islets. However, the nuclear diameter of the insulin-secreting cells was increased and correlated to the approximate body mass index (BMI) before surgery. The authors concluded that the observed hyperinsulinemia is thus just a maladaptation to the decrease in weight, and the apparent islet hyperplasia/hypertrophy represents an artifact of the preoperative insulin excess due to peripheral insulin resistance [95]. One group, however, described unrestrained β-cell proliferation from ductal progenitors in PGBHH samples [224]. According to the available literature, PGBHH is most likely a spectrum or overlap of related disorders. Therefore, different gastrointestinal anatomy; concomitant changes in incretin responses; and pancreatic changes due to preoperative obesity, which is very often associated with peripheral insulin resistance, and an overlap phase with late dumping syndrome, might best explain this syndrome. Due to the controversy about islet cell hyperplasia, surgical strategies (gastric bypass reversal or (partial) pancreatectomy) should be applied only in cases refractory to conservative treatment [389].

## 6. Epidemiology

The epidemiology of late-/adult-onset nesidioblastosis/NIPHS with hyperinsulinemic hypoglycemia is unknown. However, according to the available literature, the disease is rare. When excluding post-bariatric surgery patients and patients with diagnosed syndromes (such as MEN1), we could identify approximately 535 cases (approximately 560 when counting the “adolescent” cases reported by Castillo-López et al. [247]), although some of these cases, especially early ones and where the full text is not available/is written in another language than English/German, or demographic data are missing, are equivocal [3,5,7,11,14,15,16,17,18,19,22,23,25,26,27,29,30,31,33,34,35,39,40,41,43,45,46,47,48,49,50,52,54,55,56,57,58,59,61,62,63,66,67,68,69,70,71,73,74,77,81,82,85,87,89,91,92,94,97,98,101,104,106,108,109,110,111,112,117,121,123,125,126,129,130,131,132,134,135,136,146,147,148,149,151,152,158,159,160,164,166,170,173,175,176,178,180,181,182,184,185,186,187,192,195,196,197,198,200,203,206,208,210,211,212,214,215,216,218,219,220,221,222,225,227,228,229,230,231,232,233,237,239,240,241,242,244,247,251,256,257,259,263,266,267,269,270,272,273,275,276,277,278,279,280,281,283,285,286,293,295,296,302,305,306,307,308,314,315,316,317,318]. Many of the cases counted here overlap with the ones presented in the “Nesidioblastosis and Islet Cell Hyperplasia in other Adult Diseases” sections (e.g., the cases with concomitant insulinoma and diseases with unknown correlations to nesidioblastosis). The reporting period ranges from 1944 to 2021, and the details for every case can be seen in Supplementary Table S1. We are aware that other authors may classify the cases slightly differently. Nonetheless, no such detailed list has been provided in the literature so far. Our main criteria for counting the cases as idiopathic, late-onset nesidioblastosis were symptom onset at ≥ 3 years, hyperinsulinemic hypoglycemia, histological proof of islet hyperplasia/nesidioblastosis, a highly suggestive clinical presentation, and/or typical response to certain drugs (e.g., diazoxide/calcium channel inhibitors/somatostatin analogs). The age of the reported patients ranges from 3 to 89 years. The female:male ratio in the patients, where demographic data were available, is approximately 1.7:1, which supports the findings of Kim et al., who estimated a ratio of 1.5:1 in 2000 [54]. Unfortunately, there is, to the best of our knowledge, no definite scientific explanation for why more women are affected by the disease. It is tempting to speculate that differences in sex hormones could explain this observation. Estrogen renders cells more sensitive to insulin and reduces gluconeogenesis. This mechanism is protective concerning the onset of type 2 diabetes in premenopausal women [392,393]. However, even a mild overproduction of insulin may consequently become clinically apparent more easily in women, i.e., the likelihood increases that the blood glucose drops below values where vegetative or neuroglycopenic symptoms appear. According to our literature research, NIPHS/late-onset nesidioblastosis is thus more common than initially thought and reported by other authors. Different authors reviewed a collection of cases in the past. Stefanini et al. reported approximately 129 cases in 1974 [216]. The next largest collection was presented by Albers et al. in 1989 with a review of 20 cases [306]. Further studies with reviews of a substantial amount of cases were published later [11,46,63,74,81,128,197]. In 1995, Walmsley and colleagues suggested that nesidioblastosis accounts for approximately 0.5–5% of adult cases with hyperinsulinemic hypoglycemia [26]. Since insulinoma is already a rare disease with a yearly incidence of approximately 4 cases per 1 million persons, this would mean that nesidioblastosis occurs with a frequency of 2 to 20 cases per 100 million persons per year [394]. Service et al. found one NIPHS case among 216 insulinomas, which corresponds to approximately 0.5%. Larger populational studies were reported in 1998, 2015, and 2020. Soga et al. reviewed 1085 patients with organic hyperinsulinism from Japan over a 25-year period, of which 4.1% were diagnosed with nesidioblastosis. Approximately 2/3 of the cases had a coexisting insulinoma [40]. A Korean population was reviewed by Woo et al. in 2015 [195]. Of 84 patients with endogenous hyperinsulinism, 5 were diagnosed with nesidioblastosis. The study by Yamada et al. presented the results of a nationwide survey on endogenous hyperinsulinism in Japan in 2017–2018 [5]. Despite the relatively low response rate of adult clinics (38.2%), 205 insulinomas and 111 cases of NIPHS were reported. Among their definition of NIPHS cases, 33 were post-bariatric, 57 were classified as postprandial hyperinsulinemic hypoglycemia, 10 as nesidioblastosis, and 11 as unknown subtypes. The overall incidence of NIPHS was estimated to be 0.09/100.000 and year. Thus, with approximately 10% “genuine” nesidioblastoses, the incidence would be around 9/100.000.000 and year. This is in the range of the estimation by Walmsley and colleagues. The differentiation of postprandial hyperinsulinemic hypoglycemia and nesidioblastosis was, however, not convincingly reported. Since the large population studies are all from East Asia, ethnic differences in the incidence of the disease might be overlooked. Since there are, to the best of our knowledge, no registers counting the cases of NIPHS/nesidioblastosis, the true incidence remains unknown. The list presented in Supplementary Table S1 also only documents reported cases and is biased towards unpublished cases, and includes foremostly cases from Western countries and East Asia. We are additionally convinced that the diagnosis might be overlooked in many patients presenting with unspecific symptoms, as in our recently published case (see Dieterle et al., 2023, accepted for publication in *Biomedicines*), or might be missed at autopsy due to autolytic pancreatic tissue [395].

## 7. Symptoms

The symptoms of NIPHS/adult-onset nesidioblastosis with hyperinsulinemic hypoglycemia can be highly diverse and range from predominant cardiological to neurological or even psychiatric manifestations [15,216,226]. Our recently published case (see Dieterle et al., 2023, accepted for publication in *Biomedicines*) clearly illustrates that the clinical findings might be relatively unspecific, and only the correlation between exercising or food intake was suggestive of a metabolic disorder. Autonomic signs due to the adrenergic counterregulatory response include trembling, sweating, palpitation, tachycardia, hunger, and pallor. Neuroglycopenic symptoms can mimic many neurologic and psychiatric disorders and comprise dizziness, aphasia, visual impairment, atypical behavior, paresthesia, transient hemiplegia, psychosis, convulsion, loss of consciousness, and coma [1]. Additionally, some extraordinary manifestations of nesidioblastosis are described in the literature. Galizia et al. reported a case presenting with abdominal pain, vomiting, blurred vision, lethargy, and dehydration. Bradycardia with intermittent atrioventricular block and ST segment depression, as well as hypotension, was recognized. The patient died from sudden cardiac death due to hypoglycemia [29]. QTc changes in response to hypoglycemia are also reported in the literature. Of interest, reversible forms of hypertrophic cardiomyopathy were reported in children with CHI/PHH. The symptoms resolved after treatment of hyperinsulinism [396,397].

Weight gain was also reported in adult-onset nesidioblastosis, although it is more frequent in insulinoma [30].

Similar to our presented case, a 22-year-old patient had episodes of severe tachycardia during hypoglycemic events and reported a recurrent loss of consciousness [176].

Martin-Grace et al. described a patient who suffered from clinical symptoms of hypoglycemia 20 min after exercise onset. This is also comparable to our case [196].

An intriguing case of sensorineural deafness and recurrent facial paralysis in response to hypoglycemia is also well documented [227].

The slight hypokalemia in our presented case was retrospectively attributed to episodes of severe hyperinsulinemia. These examples demonstrate that a broad spectrum of clinical symptoms can be provoked by nesidioblastosis, despite (nearly) normal laboratory examination and a seemingly healthy patient upon office evaluation.

## 8. Clinical Diagnosis and Differential Diagnosis

The core clinical diagnostic criteria for NIPHS/adult-onset nesidioblastosis with hyperinsulinemic hypoglycemia was already cursorily discussed in Section 3. The variability in the correlation of the symptoms with food intake was also described. Fasting hypoglycemia does not exclude NIPHS, nor does postprandial hypoglycemia rule out an insulinoma [200,398]. Nonetheless, OGTT and a 72 h fasting test are the first important steps to define endogenous/organic hyperinsulinism and to document Whipple´s triad, which is characterized by a low plasma glucose (usually < 45–55 mg/dL) and clinical adrenergic and/or neuroglycopenic symptoms, which rapidly resolve upon (intravenous) glucose intake [399]. Tolbutamid stimulation of β-cells and the C-peptide suppression test were also described in the literature to demonstrate endogenous hyperinsulinism [52,216]. Subsequently, imaging studies (CT, MRI) should be performed to exclude a macroscopically detectable insulinoma. SACS of the splenic, gastroduodenal, superior mesenteric, and proper hepatic arteries can then help to detect an inadequate insulin response to calcium stimulation [34]. Additionally, SACS can support the decision on the extent of surgical intervention if planned. Coeliac angiography and percutaneous transhepatic portal venous sampling have also been applied in the past to exclude (small) insulinomas and prove the excess secretion of insulin, respectively [30]. Intraoperative insulin assays were proposed by Carneiro in 2002 to prove the successful resection of the source of insulin overproduction [69].

SACS was not performed in our present case (see Dieterle et al., 2023, accepted for publication in *Biomedicines*) since functional imaging with ^68^Ga-DOTA-Exendin-4 PET/CT showed diffuse tracer uptake in the entire pancreas, which made the diagnosis of NIPHS/nesidioblastosis highly likely. Since SACS is an invasive procedure, functional imaging techniques with high sensitivity and specificity are urgently needed to avoid the former procedure.

Some years ago, somatostatin receptor scintigraphy (Octreoscan/^111^In-pentetreotide scintigraphy; mainly targets Somatostatin receptor type 2 [SSTR2]) and ^18^F-DOPA PET were successfully used to detect focal nesidioblastosis [55,111]. Targeting of the GLP-1 receptor was first introduced in 2008 [175]. [Lys40(Ahx-DTPA-111In)NH2]exendin-4 was then used as a ligand. Subsequently, other Exendin-derived peptide tracers were developed, such as ^99m^Tc-GLP-1 or ^111^In-DOTA-Exendin-4 for planar scintigraphy/SPECT/CT, and ^68^Ga-DOTA-Exendin-4 for PET/CT [206,218,400]. The success of nesidioblastosis detection with these ligands, however, varies between studies. The GLP-1 receptor was reported not to be overexpressed in PGBHH [154,175]. Kallf et al. reported that SUV_max_ could not distinguish between NIPHS and PGBHH [237]. In idiopathic nesidioblastosis, a threefold overexpression of the GLP-1 receptor was shown histologically. Christ et al. reported a SUV_max_ of 6.9 in a patient, which is slightly lower than in our presented case (see Dieterle et al., 2023, accepted for publication in *Biomedicines*) and much lower than in typical insulinomas [206]. An important pitfall in GLP-1 imaging is the physiological uptake in Brunner´s gland in the proximal duodenum. Brunner gland hyperplasia (e.g., in association with *Helicobacter pylori* infection) was reported to cause false positive imaging (SUV_max_ of 10.0) due to the proximity to the pancreatic head [401].

Important differential diagnoses of hypoglycemia in adult patients have been briefly mentioned in the introduction section. These include drugs such as insulin, insulin secretagogues, and alcohol. In the critically ill patient, hepatic, renal, or cardiac failure, systemic inflammation/sepsis, and inanition must be considered. Hormone deficiencies (pituitary/adrenal insufficiency, glucagon deficiency) must be ruled out. Tumors producing insulin or IGFs (e.g., Doege–Potter syndrome) are another rare cause of adult-onset hypoglycemia [402]. An interesting case reported by Schröder and co-workers in 2009 describes a 54-year-old female patient with hypoglycemia and elevated IGF-1 and growth hormone levels. This constellation was later explained by an increased production of adiponectin, which was most likely due to autoimmune disease [403]. Endogenous hyperinsulinism, including autoimmune insulin hypoglycemia (antibodies against insulin or the insulin receptor; Hirata´s disease), insulinoma (or insulinomatosis with insulin expressing monohormonal endocrine cell clusters (IMECCs); a case with recurrent hypoglycemia even after total pancreatectomy was recently reported [245]), nesidioblastosis/NIPHS, and PGBHH have been discussed. Hypoglycemia factitia sometimes occurs, especially in psychiatric patients (e.g., Munchausen syndrome) [1,404].

Some attention should also be paid to the rare instances of late-onset inborn errors of metabolism presenting with hypoglycemia. Fasting hypoglycemia can occur in glycogenolysis disorders, fatty acid oxidation defects, and gluconeogenesis disorders. Postprandial hypoglycemia is associated with inherited fructose intolerance, a congenital disorder of glycosylation type Id, and late-onset forms of CHI. Exercise-induced hypoglycemia can sometimes be attributed to mutations in MCP1 (see above). A concise review of these extremely rare presentations is provided by Douillard and colleagues [405].

These remarks underscore the diagnostic challenges in adult-onset nesidioblastosis/NIPHS and the complex differential diagnostic process in adults presenting with hypoglycemia. In the corresponding Case Report (see Figure 3 in Dieterle et al., 2023; accepted for publication to *Biomedicines*), we also present a comprehensive and efficient algorithm for differential diagnosis of the non-diabetic adult patient presenting with hypoglycemia. Further clinical aspects of hypoglycemia in the adult population are discussed in the following references [1,215,402,406,407].

## 9. Therapy

The therapeutic options for adult-onset nesidioblastosis/NIPHS are limited [5,217,408]. Since the pathophysiology of the disease is still barely understood, interventions that causally address the functional β-cell disorder have not been developed.

The first step is usually to try a low-carbohydrate diet with a low glycemic index to avoid strong insulin responses. This scheme is, however, mostly not successful. Pharmacological treatment relies on α-glucosidase inhibitors (Acarbose, Voglibose) [98], the ATP-sensitive potassium channel agonist diazoxide, calcium channel antagonists (Verapamil, Amlodipine, Nifedipine) [63,116,145], and somatostatin analogs (octreotide, lanreotide, pasireotide) [309]. Except for acarbose, which slows down carbohydrate resorptions, all other drugs aim at indirectly reducing insulin secretion from the islets of Langerhans. Unfortunately, these treatments are often associated with intolerable adverse effects. Apart from fluid retention, diazoxide can rarely cause severe symptoms such as angina pectoris or even myocardial infarction, as seen in our patient (see Dieterle et al., 2023, accepted for publication in *Biomedicines*). Calcium channel inhibitors lead to hypotension and can significantly impair the quality of life.

The different somatostatin analogs have distinctive affinities for the various somatostatin receptors. While octreotide mainly acts through SSTR2, pasireotide has a high affinity for SST5, which contributes to the explanation of different clinical effects [213]. Of note, somatostatin analogs, especially octreotide, also reduce glucagon secretion, which can lead to an exacerbation of hypoglycemic events [196]. This was also the case with our patient (see Dieterle et al., 2023, accepted for publication in *Biomedicines*).

In some instances, glucocorticoids, β-blockers such as propranolol, or antipsychotic/antiepileptic drugs such as phenytoin were also used in the treatment of NIPHS/nesidioblastosis since these drugs induce hyperglycemia [15]. Everolimus was also tried as a treatment in one patient but failed to establish euglycemia [213] (Table 1).

In PGBHH, feeding via a gastric tube was successful in some patients [410]. Pharmacological blocking of the GLP-1 receptor with exendin derivates could also mitigate disease symptoms in preliminary studies [411,412].

In many patients, partial pancreatectomy or even subtotal/total pancreatectomy remains the only option to control the symptoms adequately. The extent of resection is still a matter of debate. While some prefer limited pancreatic resection (50–60%), others suggest subtotal pancreatectomy (80–95%), as usually performed in children suffering from CHI [89,135]. In a subset of patients, symptoms recur after partial/distal pancreatectomy, making completion pancreatectomy necessary, as seen in our patient (see Dieterle et al., 2023, accepted for publication in *Biomedicines*) and others [87]. The surgical intervention is, however, associated with considerable postoperative morbidity (diabetes mellitus type 3c, exocrine pancreatic insufficiency) and mortality. Thus, surgery should be performed in high-volume centers only [413,414]. In PGBHH, reversal of the bypass or pancreatic resection has been successfully used [165,174].

Since these treatment options are somewhat unsatisfactory, innovative, and less invasive strategies are needed. Boss et al. presented an experimental study evaluating a receptor-targeted photodynamic therapy for GLP-1 receptor-positive lesions [415]. Exendin-4 was coupled to a photosensitizer, and interstitial irradiation of the molecule led to the selective killing of GLP-1 receptor-positive cells in a hamster model. While endocrine pancreatic cells (derived from a rat insulinoma cell line or hamster lung cells transfected with GLP-1 receptor) underwent apoptosis, exocrine pancreatic cells (human pancreatic tumor cell line) survived, showing the specificity and selectivity of this approach. Such techniques or peptide-receptor radionuclide therapies directed against the GLP-1 receptor could be used in the future to overcome current therapeutic challenges. Thus, promising options for a selective reduction in β-cell mass without the need for surgical intervention are under development.

The gender-specific efficiency of the different drugs currently available for adult-onset nesidioblastosis/NIPHS should be further evaluated [416]. To the best of our knowledge, there is currently no evidence that there are sex differences in the response of NIPHS to the different treatment options. However, such differences were reported for several drugs, such as GLP-1 agonists in diabetes therapy, where therapeutic effects seem to be more favorable in women due to hormonal mechanisms [417]. This is of interest in the context of NIPHS/PGBHH since the GLP-1 signaling system is potentially involved in the pathogenesis of the disease. Sex-specific differences in drug action have also been reported for somatostatin analogs in the in vivo rat models of pituitary tumors. The authors report that the differential expression of somatostatin receptor subtypes may be responsible for these observations and could also be of relevance for clinical application [418]. Therefore, this interesting field of study should be further evaluated in the context of NIPHS/adult-onset nesidioblastosis to avoid unnecessary surgical interventions.

**Figure 2:**
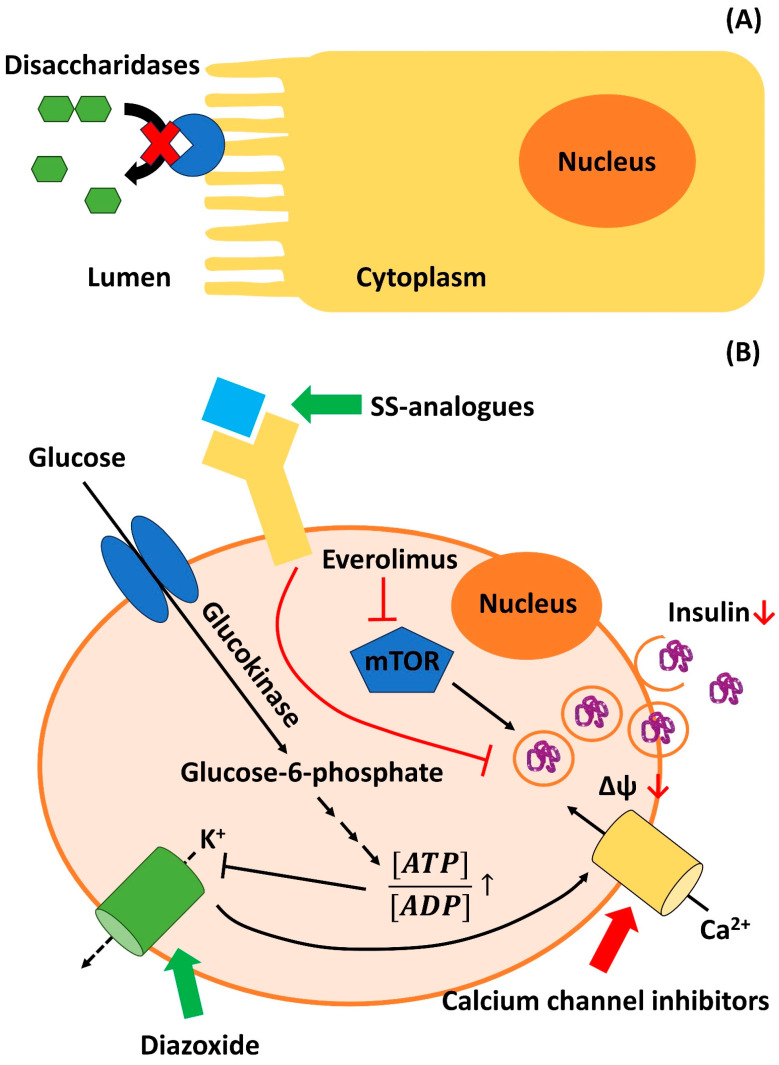
A selection of current pharmacological treatment options for adult-onset nesidioblastosis/NIPHS with the corresponding mechanisms of actions: (**A**) α-Glucosidase inhibitors such as Acarbose inhibit (red cross) intestinal disaccharidases (blue enzyme) in the cuticular layer of enterocytes. Thus, disaccharides such as maltose (connected green hexagons) cannot be hydrolyzed into monosaccharides. This, in turn, reduces the resorption of carbohydrates and the corresponding insulin response. (**B**) Other pharmacological compounds directly act on molecular targets on/within the pancreatic β-cells. Diazoxide stimulates (green arrow) K_ir_6.2/K_ATP_ potassium (K^+^) channels (green barrel), which stabilizes the resting membrane potential (Δψ) of the β-cells. Calcium (Ca^2+^) channel inhibitors such as Verapamil or Nifedipine block (red arrow) the activity of voltage-gated calcium channels (yellow barrel), which prevents the depolarization of the endocrine cells. Somatostatin (SS) receptors (yellow Y-structure) are G-protein coupled receptors and can be subdivided into different isoforms (see main text). Upon binding of somatostatin or somatostatin analogs such as octreotide, lanreotide, or pasireotide (blue rectangle), which activate (green arrow) SS receptors, intracellular downstream signaling cascades of the SS receptors (details are exemplarily given in [419,420]) result in an inhibition (red arrow) of insulin release. Inhibitors (red arrow) of mammalian targets of rapamycin (mTOR; blue pentagon), such as Everolimus, also lead to a decrease in insulin synthesis and secretion through complex mechanisms (for details, see [421]).

The current therapeutic options for adult-onset nesidioblastosis/NIPHS are summarized in Table 1 and visually illustrated in Figure 2.

## 10. Conclusions and Future Perspectives

In this review article, we presented a comprehensive and in-depth discussion of diffuse adult-onset nesidioblastosis/NIPHS, a rare cause of hyperinsulinemic hypoglycemia. Due to clinical, epidemiological, and histopathological features, we propose that “late-onset islet dysplasia/islet cell atypia with NIPHS” would be an adequate description of the disease. When applying strict criteria, only some hundred cases of this rare disease have been described in the international medical literature within the last 100 years. However, it can be assumed that this entity is often overlooked. Patients repeatedly report a long history of seeking medical help and suffer from unbelievable constraints in their daily activities. A complex clinical presentation, in combination with the different ages of onset, makes it difficult to diagnose the condition clearly. Since conservative treatment options are limited, many patients undergo partial or total resection of the pancreas, which results in considerable morbidity and impairment in the quality of life. Thus, innovative and less invasive treatment options are needed in the future, which aim at preserving the exocrine and endocrine pancreatic function while selectively reducing or inhibiting the insulin-secreting β-cells. This, however, depends on a clear pathophysiological understanding of the disease. While there is some convincing experimental evidence for functional dysregulation of β-cells, differences in clinical and histopathological appearances of the disease cannot be explained currently. Clear differentiation from PGBHH is also necessary since different pathogenesis is suspected.

We recently also submitted an illustrative Case report of a 23-year-old man presenting with this exciting disease, and the interested reader is directed to this article (Dieterle et al. 2023, accepted for publication in *Biomedicines*). 

## Figures and Tables

**Table 1:** Therapeutic options for adult-onset nesidioblastosis/NIPHS.

Therapeutic Principle	Mechanism of Action ^1^	Therapeutic Effect Based on the Current Literature ^1^
Low carbohydrate diet/diet with low glycemic index	Limits insulin secretion postprandially due to low slope of glucose increase after food intake	Low but non-invasive
α-glucosidase inhibitors (Acarbose, Voglibose)	Slows down glucose resorption through inhibition of carbohydrate-digesting enzymes in the intestines	Low but non-invasive; few adverse effects
Diazoxide	Activates ATP-dependent potassium channels in β-cells → stabilizes resting membrane potential → inhibits insulin secretion	Sometimes effective; may have severe adverse effects (fluid retention, angina pectoris)
Calcium-channel antagonists (Verapamil, Amlodipine, Nifedipine)	Inhibit voltage-dependent calcium channels → inhibit depolarization of β-cells → inhibit insulin secretion	Sometimes effective; may have severe adverse effects (hypotension)
Somatostatin analogs (octreotide, lanreotide, pasireotide)	Stimulate somatostatin receptors (G-protein coupled receptors) on β-cells → inhibit insulin secretion	Sometimes effective; may lead to increased frequency/intensity of hypoglycemia (due to inhibition of glucagon; depends on receptor specificity)
Glucocorticoids	Induce gluconeogenesis and glycogenolysis in the liver; augment effects of glucagon; induce peripheral insulin resistance	Sometimes effective; long-term treatment associated with severe adverse effects
β-blockers (e.g., propranolol)	Mechanism not entirely clear (β1-adrenoceptor-mediated inhibition of insulin secretion?)	Rarely effective; β-blockers also tend to precipitate hypoglycemia (especially through inhibition of β2-adrenoceptor-dependent glycogenolysis)
Antipsychotics/Antiepileptics (e.g., phenytoin)	Probably through insulin insensitivity	Rarely effective; may have severe adverse effects
Everolimus	Inhibition of mammalian target of rapamycin (mTOR) signaling, which is involved in the regulation of insulin secretion	Rarely effective (more likely to be effective in the pediatric population [409])
(Sub)total pancreatectomy	Surgical removal of the islets of Langerhans	Effective, if enough endocrine tissue is removed; considerable morbidity and mortality
Receptor-targeted photodynamic therapy	Selective destruction of GLP-1 receptor-expressing cells	Experimental treatment (animal model)

^1^ Details and references are given in the main text.

## Data Availability

The data presented in this study are available in Supplementary Table S1.

## Supplementary Materials

The following supporting information can be downloaded at: https://www.mdpi.com/article/10.3390/biomedicines11061732/s1, Supplementary Table S1. Overview of cases with adult-onset Nesidioblastosis/NIPHS.
