# Supplementary material for: Diffuse, Adult-Onset Nesidioblastosis/Non-Insulinoma Pancreatogenous Hypoglycemia Syndrome (NIPHS): Review of the Literature of a Rare Cause of Hyperinsulinemic Hypoglycemia
Source: Biomedicines. 2023 Jun 16;11(6):1732. doi: 10.3390/biomedicines11061732 (PMC10296556; doi:10.3390/biomedicines11061732)
Supplement: Supplementary file 1 [file biomedicines-11-01732-s001.zip › biomedicines-2439483-supplementary.pdf]

Number	Author	Year	Cases	(Special) feature	Pathologic features pancreas	Clinical symptoms/laboratory findings	Therapy	Evaluation	Literature
									*full text not available
1	Lang	1925	1		nodular hyperplasia of islets ("adenomatosis")			unclear, if case of NIPHS/nesidiobalstosis and adult*	Cited in Frantz, 1944
2	John	1931	1	Diabetes, Hyperthyroidism, Cirrhosis of the liver, liver/gallbladder carcinoma interstitial pancreatitis; insulin discontinued	atrophy and hypertrophy of islets	hypogylcemia (30 mg/dL)		unclear, if case of NIPHS/nesidiobalstosis and adult; alternatively hyperplasia due to pancreatitis and hypoglycemia due to liver failure*	Cited in Frantz, 1944
3	Frantz	1944	11	Report of different cases of insulin producing neoplasms/adenomas/adenomatosis; among them 11 cases (10 aged > 16; 1x girl with 7 year) with hypertrophy and/or hyperplasia of islets of Langerhans without neoplasm	islet hyperplasia or hypertrophy or number of islets increased	hypoglycemia reported for all patients	some: partial Pancreatectomy	possible cases of NIPHS/nesidiobalstosis	
4	Summerskill et al.	1959	1	jejunal ulceration, malabsorption, hypergastrinemia	islet cell hyperplasia			unclear, if adult*	Cited in Creutzfeldt, 1975
5	Friedlander	1960	22	6 with history of chronic pancreatitis	islet cell hyperplasia	hyperinsulinism		possible cases of NIPHS/nesidiobalstosis; unclear, if all adult cases*	Cited in Sandler, 1975
6	Franksson et al.	1960	1	chief-cell hyperplasia of parathyroid; adenomata pancreas, gastro-duodenal ulcers; possibly MEN1 with gastrinoma	islet cell hyperplasia			unclear, if adult; possible nesidioblastosis in MEN1*	
7	Bloodworth	1963	34	post mortem analysis; all patients treatment with tolbutamid (sufonyl urea)	islet hyperplasia, neo formation of islets, increase in α cells, decrease in β cells			changes associated with sulfonylurea therapiy; unclear if all patients affected from the changes*	
8	Ellison et al.	1964	26	all Zollinger-Ellison syndrome patients	islet cell hyperplasia			unclear, if all adult (but many)	
9	Knight	1967	1	Insulinoma, Diabetes mellitus	generalized islet cell hyperplasia			unclear, if adult; possible background nesidioblastosis in insulinoma*	
10	Cavallero et al.	1967	2	Zollinger-Ellison syndrome	islet hyperplasia	not reported		no patient characteristics reported	
11	Paloyan et al.	1967	1	69-yr-old man; calcified pancreatitis, hyperparathyroidism	islet cell hyperplasia (A cell excess)				Cited in Ouyang, 2011
12	Brown et al.	1968	1	Associated with Zollinger-Ellison syndrome	islet hyperplasia		Resection	unclear, if adult*	
13	Vance et al.	1969	6	Family with multiple endocrine adenomatosis, hyperinsulinism		hyperinsulinism		unclear, if all adult, unclear if pathological examination*	
14	Sircus et al.	1970	1	water-losing, electrolyte-losing diarrhoea	mild hyperplasia of islet tissue, overrepresentation of beta cells		Partial resection pancreas	possibly VIPoma with islet cell hyperplasia	
15	Brown et al.	1971		associated with cystic fibrosis	nesidioblastosis			*	Cited in Jabri, 2004
16	Hight et al.	1971	1	53-yr-old man; duodenal ulcer despite vagotomy/Pyloroplasty hypergastrinemia (Zollinger-Ellison-Syndrome)	increased number islet cells	hyperacidity, ulcer	Partial resection pancreas	Zollinger-Ellison-Syndrome with islet cell hyperplasia*	
17	Greene et al.	1972	1		islet cell hyperplasia			unclear, if adult*	
18	Jacobs et al.	1972	1	wateray diarrhea and hypokalemia	non-β islet cell hyperplasia			unclear, if adult; possibly VIPoma with islet cell hyperplasia	
19	Vance et al.	1972	4	3 patients from a family with familial multiple endocrine adenomatosis (=MEN1); 1 non-family member suspected to have multiple endocrine adenomatosis (= MEN1) patients in study aged 17-72 years; excessive insulin secretion	islet cell hyperplasia	hypoglycemia, not all patients symptomatic	Diazoxide or Pancreatectomy	islet cell hyperplasia	
20	Larsson et al.	1973	1	Associated with gastrinoma	increased number of islets, ductulo-insular complexes	recurrent duodenal ulcers	Resection	unclear, if really adult*	
21	Creutzfeldt et al.	1975	5	Zollinger-Ellison syndrome patients	islet cell hyperplasia			unclear, if all adult	
22	Verner et al.	1974	11	watery diarrhoea syndrome; at least 11 of 54 reported cases only diffuse islet cell hyperplasia	diffuse non-β islet cell hyperplasia		total pancreatectomy	possibly VIPoma with islet cell hyperplasia*	Cited in Lesna, 1977
23	Stefanini et al.	1974	129	series of many communications/thorough review of literature	islet cell hyperplasia	all patients with hypoglycemia	corpocaudal/subtotal pancreatectomy	description of cases a bit unclear; approx. 129 adult cases	
24	Jahnke et al.	1975	1	sensorineural deafness, recurrent facial paralysis (unilateral)	islet cell hyperplasia	hypoglycemia	Subtotal pancreatectomy	unclear, if adult; possible NIPH/nesidioblastosis*	
25	Rastogi et al.	1975	1	study with administration of insulin secretagogues/glucose/amino acids/ glucagon				probably islet cell hyperplasia*	
26	Sandler et al.	1975	1	insulin-dependent Diabetes mellitus; later developing fasting hypoglycemia: 59-yr-old man; introducing C-peptide for measuring endogenous hyperinsulinism	islet cell hyperplasia, neoproliferation of islet cells (ductulo-insular complexes)	fasting hypoglycemia	2/3 pancreatectomy	Subtotal pancreatectomy	
27	Bradley et al.	1976	1	48-yr-old male	islet cell hyperplasia	hypoglycemia	95% pancreatectomy		Cited in Martignoni, 2003
28	Mendelsohn	1976	1	kidney transplant recipient; multiple malignancies, 54-yr-old man, autopsy	islet cell hyperplasia			unclear significance*	
29	Larsson et al.	1977	14	different diseases ranging from insulinoma, to glucagonoma, and Zollinger-Ellison syndrome; as well as pernicious anemia and atrophic gastritis	different "types" of islet cell hyperplasia and/or nesidioblastosis	some with hyperinsulinism		unclear, if all adult as age not reported	
30	Greider et al.	1977	9	unusual perinuclear inclusions	islet cell hyperplasia		Partial pancreatectomy	unclear significance; unclear, if any adult cases*	
31	Lesna et al.	1977	1	39-yr-old man; laxative abuse; diarrhea	increased number of islets; neoformation islets; ductulo-insular complexes				
32	Ingemansson et al.	1977	1	32-yr-old woman; detection by pancreatic vein catheterization	localized islet cell hyperplasia	hyperinsulinemic hypoglycemia	distal Hemipancreatectomy		
33	Hayashi et al.	1977	1	11 -yr-old girl	nesidioblastosis	fasting hypoglycemia and hyperinsulinism	80% Resection	unclear, if really adult (11-yr-old)	
34	Efendic et al.	1978	1	Somatostatin treatment of hyperinsulinism	islet cell hyperplasia			possibly NIPHS/nesidioblastosis; unclear, if adult*	
35	Schwartz et al.	1978	1	secretory diarrhea with VIP level increase	islet cell hyperplasia			unclear, if really adult*	
36	Schikman et al.	1978	1	10-yr-old boy	islet cell hyperplasia	fasting hypoglycemia		unclear, if really adult (10-yr-old)*	
37	Ingemansson et al.	1978	2		islet cell hyperplasia	organic hypoglycemia		unclear, if really adult*	
38	Kidd et al.	1979	1	46-yr-old woman; watery diarrhea, hypokalemia, hypochlorhydria	diffuse pancreatic islet cell hyperplasia		75% pancreatectomy	(pseudo)VIPoma with islet cell hyperplasia	
39	Varas Lorenzo et al.	1979	1	58-yr-old, hypergastrinemia, recurrent ulcers	islet cell hyperplasia		subtotal pancreatectomy	Zollinger-Ellison-Syndrome with islet cell hyperplasia*	
40	Dahms et al.	1980	6	3 to 15-yr-old patients (additional 10 patients with nesidioblastosis and hyperplasia, who all presented with symptoms in the first year of life)	nesidioblastosis but no hyperplasia (5 patients; defined as scattered islet cells throughout acinar tissue); some with additional ductulo-insular complexes; nesidioblastosis + islet cell hyperplasia (1 patient)	hyperinsulinism (not reported in all)	Subtotal pancreatectomy	unclear, if really "adult"	
41	Tomita et al.	1980	2	one with watery diarrhea, one without	pancreatic polypeptice cell hyperplasia, large/atypical islets			unclear, if really adult*	
42	Friesen et al.	1980	6	6 patients in a series with 26 patients	islet cell hyperplasia with or without nesidioblastosis		some with distal pancreatectomy	unclear, if really adult (age not reported)	
43	Leong et al.	1980	1	associated with metastasizing insulinoma, multiple adenomas, 40-yr-old woman	hyperplasia of islets, nesidioblastosis		75% resection	possible case of background nesidioblastosis in insulinoma	
44	Brennan et al.	1980	1	59-yr-old woman; biliary cirrhosis, gastrojejunostomy, portocaval anastomosis	islet hyperplasia, increase in number of islets	hypoglycemia, hyperinsulinism, hyperglucagonemia	pancreatic resection	possibly NIPHS/nesidioblastosis secondary to gastrectomy	
45	Reichardt et al.	1980	?	report of cases of islet cell hyperplasia in selective vein catheterization study		organic hyperinsulinism		unclear, how many (adult) cases*	
46	Glaser et al.	1981	2	20-yr-old male and 58-yr-old female	1x islet cell hyperplasia with macroadenoma, 1x nesidioblastosis	organic hyperinsulinism	operation		
47	Duncan et al.	1981	1	22-yr-old woman	islet cell hyperplasia	symptomatic hypoglycemia	near-total pancreatectomy		
48	Bonfils et al.	1981	13	Zollinger-Ellison syndrome	Islet cell hyperplasia		distal pancreatectomy	not reported, if all adult	
49	Nathan et al.	1981	1	58-yr-old woman	abnormal shape and size of islets, increased number, ductulo-insular complexes	fasting hypoglycemia and hyperinsulinism	75% Resection	*	Cited in Martignoni, 2003
50	Harness et al.	1981	6	11-57 years old patients	similar findings like in adults	hypoglycemia (?)	50-100% Resection	unclear, if all cases really "adult"*	Cited in Martignoni, 2003
51	Bartow et al.	1981	20	Autopsy study on patients with pancreatic fibrosis	(minimal) nesidioblastosis			nesidioblastosis interpreted as variant/normal/mildly abnormal stimulation;not clear, if all adult*	Cited in Karnauchow, 1982
52	Jennette et al.	1982	1	scleroderma, anti-insulin receptor antibody induced diabetes mellitus	islet cell hyperplasia	no hypoglycemia		unclear, if really adult; thought to compensate for autoantibody-syndrome*	
53	Cho et al.	1982	2		1x islet cell hyperplasia with adenoma, 1x nesidioblastosis	hyperinsulinism			
54	Karnauchow	1982	76	Autopsy study of 207 adults	(minimal) nesidioblastosis (defined by ductulo-insular complexes)	no clinical signs of hyperinsulinism/diabetes or pancreatic tumors		nesidioblastosis interpreted as variant/normal/mildly abnormal stimulation	

55	NEJM Case Reports	1983	1						Cited in Service, 1999
56	Weidenheim et al.	1983	5	29-56-yr-old; hypoglycemia in some related to exercise; one case with additional insulinoma	nesidioblastosis (defined as islet cells scattered throughout acinar tissue)/islet cell hyperplasia	hyperinsulinemic hypoglycemia	all partial pancreatectomy		
57	Zhu et al.	1983	1	concomitant insulinoma	islet cell hyperplasia		reoperation	unclear, if really adult*	
58	Oliver et al.	1983	1	32-yr-old woman; MEN1 syndrome with pancreatic carcinoma	nesidioblastosis	hyperinsulinemic hypoglycemia		*	
59	Keller et al.	1983	1	47-yr-old woman	increase in number of islets, size variability, aggregated islets, ductulo-insular complexes	hyperinsulinemic hypoglycemia	distal pancreatectomy + Diazoxide		
60	Rayman et al.	1984	1	25-yr-old woman; chlorpropamide-induced (sulphonylurea)	islet cell hyperplasia and nesidioblastosis	hyperinsulinemic hypoglycemia	Subtotal pancreatectomy		
61	Bauman et al.	1984	1	26-yr-old woman	nesidioblastosis	hyperinsulinemic hypoglycemia	surgical intervention	*	
62	Gould et al.	1984	2	29 and 63-yr-old woman	"adult nesidiodysplasia"		partial pancreatectomies	*	Cited in Albers, 1989
63	Harrison et al.	1984	3	6 patients with either islet cell hyperplasia or nesidioblastosis; 3 of them already reported by Harness, 1981	2x islet cell hyperplasia, 1x nesidioblastosis	hyperinsulinemic hypoglycemia	distal pancreatectomy		
64	Tomita et al.	1985	1	66-yr-old man; watery diarrhea, hypokalemia, achlorhydria	islet cell hyperplasia, mainly pancreatic polypeptide and glucagon cells		surgery		
65	Bani et al.	1985	2	both patients with additional insulinoma	nesidioblastosis (defined as islet cells scattered throughout acinar tissue); A and B cells; also intermediate cells (acinar-islet cells) regarded as neoformation of islets	hyperinsulinemic hypoglycemia		possibly insulinoma with background nesidioblastosis*	
66	Bani Sacchi et al.	1985	4	all patients with hypergastrinemia	increased number of islets; nesidioblastosis (defined as islet cells scattered throughout acinar tissue); many A and B cells; increase in islet size, irregular islet shapes			unclear, if really adult*	
67	Campbell et al.	1985	1	36-yr-old man; additional insulinoma; islet cell surface autoantibodies present; higher proliferative capacity of islet proliferation (in vitro) of specimens from nesidioblastosis patient	islet cell hyperplasia, ductal proliferation and budding; B-cell degranulation	hyperinsulinemic hypoglycemia	partial pancreatectomy	possibly insulinoma with background nesidioblastosis	
68	Madeira et al.	1986	1	36-yr-old woman, additional small insulinoma	nesidioblastosis (ductulo-insular complexes)	fasting hypoglycemia	total pancreatectomy	possibly insulinoma with background nesidioblastosis	
69	Illyés et al.	1986	1		nesidioblastosis			*	
70	Klöppel et al.	1986	3	case series of nine adult patients with MEN1	nesidioblastosis (as ductulo-insular complexes), no islet cell hyperplasia	some with hypoglycemia	partial pancreatectomies	suggested that nesidioblastosis is not a reliable criterion for MEN1	
71	Weinstock et al.	1986	1	32-yr-old man	islet cell hyperplasia	hyperinulinemic hypoglycemia; elevation of fasting insulin	50% pancreatectomy		
72	Ray et al.	1986	5	adult patients with genetic alpha 1-Antitrypsin deficiency	islet cell hyerplasia; some with nesidioblastosis				
73	Kovacs et al.	1986	1	30-yr-old woman	nesidioblastosis and peliosis of pancreatic islets			*	
74	Odaira et al.	1987	1	localized pancreatitis, obstructive pancreatic duct, endocrine tumor	nesidioblasotis in the pancreatic part with obstruction			unclear, if adult; unclear if related to the endocrine tumor or to the pancreatitis/obstruction*	
75	Jerkins et al.	1987	1	63-yr-old man, medullary thyroid carcinoma, watery diarrhea, flushing	pancreatic nesidioblastosis and microadenosis (PP hypersecretion)		partial resection + somatostatin analogue	suspected to be overlap syndrome of MEN1 and MEN2	
76	Carlson et al.	1987	1		nesidioblastosis	persistent hypoglycemia		*	
77	Asa et al.	1987	1	30-yr-old woman; MEN1, GHRH-producing tumor, hyperparathyroidism, hyperprolactinemia, multiple endocrine pancreatic tumors	nesidioblastosis	hypoglycemia	Subtotal pancreatectomy	*	
78	Glaser et al.	1988	3	2x adult with B-cell adenomas and islet hyperplasia (aberrant reaction to secretin stimulation of insulin release; independent of MEN1); 1x with nesidioblastosis (normal response to secretin stimulation; symptoms from 2 years on)	nesidioblastosis or islet cell hyperplasia	hypogylcemia	subtotal pancreatectomy		
79	Roncari et al.	1988	1	25-yr-old woman; more rapid growth of the islets cells in culture than normal cells	islet cell hyperplasia and adenomatosis	hyperinsulinemic hypoglycemia	2/3 pancreatectomy		
80	Kohnert et al.	1988	1		nesidioblastosis			unclear, if adult*	
81	Balas et al.	1988	1	35-yr-old white woman, tumor in head of pancreas, cholecystectomy, additional (silent) glucagonoma	nesidioblastosis, scattered A cells throughout acinar tissue		pancreatectomy	likely first report of nesidioblastosis associated with glucagonoma	
82	Lamberts et al.	1988	1		nesidioblastosis		somatostatin analogue	unclear, if really adult*	
83	Woltering et al.	1988	1		nesidioblastosis		somatostatin analogue	*	
84	Derizhanova	1989	1		nesidioblastosis			likely adult*	
85	McHenry et al.	1989	3	3 women (29, 42, 63-yr-old)	nesidioblastosis (neoformation of islets from ducts)	hyperinsulinemic hypoglycemia	near-total pancreatectomy		
86	Sawady et al.	1989	1	Patient with Zollinger-Ellison syndrome	islet cell hyperplasia			*	
87	Risaliti et al.	1989	1	hypertension, choristoma	nesidioblastosis of heterotopic pancreas			*	
88	Chines et al.	1989	1		nesidioblastosis	hyperinsulinism		*	
89	Albers et al.	1989	1	43-yr-old woman	nesidioblastosis, ductulo-insular complexes, islet apposition to ducts, islet cell hypertrophy, islet enlargement	hyperinsulinemic hypoglycemia	75% pancreatectomy		
90	Fong et al.	1989	3		nesidioblastosis, diffuse islet cell hyperplasia	hyperinsulinemic hypoglycemia		*	Cited in Martignoni, 2003
91	Fajans et al.	1989	15		nesidioblastosis	hyperinsulinemic hypoglycemia		unclear, if (all) adult*	Cited in Carneiro, 2002
92	Martinez Valls et al.	1990	2	45 and 73-yr-old males	nesidioblastosis	hyperinsulinemic hypoglycemia	subtotal pancreatectomies	*	
93	Mozell et al.	1990	1	clinical nesidioblastosis		hyperinsulinemic hypoglycemia	somatostatin analogue	*	
94	Alhindawi et al.	1990	1	52-yr-old woman, secretory diarrhea	islet cell hyperplasia			*	
95	Kohnert et al.	1990	?	histological study				unclear, if adult*	
96	Bani et al.	1991	1	malignant glucagonoma	nesidiblastosis (scattered B cell throughout pancreas parenchyma)			*	
97	Toda et al.	1991	1	with malignang microglucagonoma	islet cell hyperplasia (A cell excess)			*	Cited in Ouyang, 2011
98	Kimura et al.	1991	5	5 patients from an autopsy study of 800 adults showed islet cell hyperplasia	hyperplasia of islets of Langerhans	no clinical symptoms documented		*	
99	Böttger et al.	1992	1	concomitant adenomatosis	nesidioblastosis	organic hyperinsulinism		*	
100	Burman et al.	1992	2	adult onset hyperinsulinism in two siblings	islet cell hyperplasia	organic hyperinsulinism		*	
101	Andrews et al.	1992	1	60-yr-old male; concomitant insulinoma	nesidioblastosis (defined as diffuse hyperplasia + apparent budding of islets from pancreatic ducts)	hyperinsulinemic hypoglycemia	50% distal pancreatectomy	possibly insulinoma with background nesidioblastosis	
102	Tibaldi et al.	1992	1	concomitant adenomatosis of the pancreas	ß-cell islet cell hyperplasia but no nesidioblastosis	postprandial hypoglycemia, no fasting hypoglycemia	distal pancreatectomy	possibly insulinoma with background nesidioblastosis/hyperplasia*	
103	Chen et al.	1993	2	43-yr-old woman (insulinoma resected years ago), 52-yr-old female	diffuse islet cell hyperplasia, ductulo-insular complexes, termed "nesidiodysplasia"	hypoglycemia	75-80% pancreatectomy	one case possibly insulinoma with background nesidioblastosis*	
104	Gaulier et al.	1993	1	58-yr-old woman with insulinoma, chronic pancreatitis, villous adenomatosis of pancreatic duct	islet cell hyperplasia (B, A and D cells)	hypoglycemia	pancreatectomy	possibly insulinoma with background nesidioblastosis*	
105	Tavcar et al.	1994	?			hyperinsulinemic hypoglycemia		*	
106	Geoghegan et al.	1994	2		islet cell hyperplasia	hyperinsulinemic hypoglycemia	distal pancreatectomy	unclear, if really adult*	
107	Farley et al.	1994	2		pancreatic nesidioblastosis with gastrin/PP (pancreatic polypeptide) secretion		pancreatectomy	*	
108	Losada et al.	1995	1		nesidioblastosis	hyperinsulinemic hypoglycemia		*	Cited in Martignoni, 2003
109	Lechleitner et al.	1995	1	young woman	nesidioblastosis	hyperinsulinemia		*	
110	Walmsley et al.	1995	1	84-yr-old woman	diffuse nesidioblastosis	hyperinsulinemia	pancreatectomy	*	
111	Bell et al.	1995	1	diabetes mellitus reversed by nesidioblastosis	nesidioblastosis	hyperinsulinemic hypoglycemia		unclear, if really adult*	
112	Le Bodic et al.	1996	9	9 of 28 MEN1 patients had nesidioblastosis but no islet cell hyperplasia (16- to 57-yr old; 17 male, 11 female)	nesidioblastosis but no islet cell hyperplasia	8 patients with hyperinsulinemia; all with hypoglycemia (of the whole study population)	surgery		
113	Galizia et al.	1996	1	40-yr-old man, predominant cardiac manifestations; sudden death	increase in number and size of islets; nesidioblastosis (autopsy specimen)	hyperinsulinemic hypoglycemia	death (arrhythmia?)		
114	Kim et al.	1996	1	50-yr-old man, repeated loss of consciousness	hyperplasia of islets (predominantly pancreatic head); increased in size and number; ductulo-insular complexes; dysplastic changes: increased nuclear-cytoplasmic ratio, hyperchromatism, coarse chromatin, prominent nucleoli; increase in total endoccrine area (7% in head); hyperplasia more pronounced in pancreatic head	hyperinsulinemic hypoglycemia	70% proximal pancreatic resection		
115	Fuller et al.	1997	1	24-yr-old woman	nesidioblastosis	hyperinsulinemic hypoglycemia	subtotal distal pancreatectomy + postoperative diazoxide		
116	Martella et al.	1997	3	3 adult women; all with gastrinomas (pancreatic/duodenal)	islet cell hyperplasia (PP expressing cells)		surgical resection		
117	Garcia et al.	1997	1	30-yr-old woman	diffuse nesidioblastosis; varying number and size of islets; diffuse proliferation, ducto-endocrine proliferation; irregular/large islets; nuclear variability (size/staining)	hypoglycemia	subtotal pancreatectomy		

118	Lee et al.	1997	1	69-yr-old woman	diffuse nesidioblastosis	hyperinulinemic hypoglycemia	subtotal pancreatectomy	*	
119	Röher et al.	1997	1	18-yr-old man	islet cell hyperplasia	hyperinsulinemic hypoglycemia	subtotal pancreatectomy		
120	Sanjuan Portugal et al.	1997	1		nesidioblastosis			*	
121	Conget et al.	1997	1		nesidioblastosis	no clinical findings			
122	Brown et al.	1998	2	with glucagonoma	A cell hyperplasia	diabetes		*	
123	Ueda et al.	1998	1	23-yr-old man	islet cell hyperplasia, nesidioblastosis; normal proportion insulin/glucagon/somatostatin positive cells	hyperinsulinemic hypoglycemia	75% pancreatectomy		
124	Soga et al.	1998	44	Large studygroup from Japan with 1085 cases of organic hyperinsulinism; 4.1% of patients with nesidioblastosis (2.6% with concomitant insulinoma; 1.6% without)	nesidioblastosis	hyperinsulinemic hypoglycemia		unclear, if all adult*	
125	Pereira et al.	1998	1	46-yr-old woman	"nodular hyperplasia" (focal and unencapsulated)	hyperinsulinemic hypoglycemia	partial pancreatectomy		
126	Rinker et al.	1998	1		nesidioblastosis	hypoglycemia	Diazoxide	*	
127	Glaser et al.	1998	5	2- 36-yr-old; activating Glucokinase mutations (familial)		hyperinsulinemic hypoglycemia	Diazoxide		
128	Pasieka et al.	1999	1	37-yr-old woman; watery diarrhea	islet cell hyperplasia (PP expressing cells)		surgery		
129	Service et al.	1999	5	all with exclusively postprandial hypoglycemia but negative fasting test, 16-78-yr-old (4 men/1 woman)	islet hypertrophy and nesidioblastosis	hyperinsulinemic hypoglycemia	partial pancreatectomy		
130	Eriguchi et al.	1999	2	34- and 39-yr-old women, fainting, nausea, loss of consciousness	graded slight hyperplasia of islets	hyperinsulinemic hypoglycemia	distal pancreatectomy		
131	Wängberg et al.	1999	1		nesidiodysplasia	hyperinsulinemic hypoglycemia	95% distal pancreatectomy	*	
132	Tomaszewska et al.	1999	1	66-yr-old man; chronic alcohol-induced pancreatitis	nesidioblastosis	hyperinsulinemic hypoglycemia	subtotal pancreatectomy	*	
133	Park et al.	1999	?		nesidioblastosis	hyperinsulinemic hypoglycemia		unclear, if all adult*	Cited in Woo, 2015
134	Yeh et al.	1999	1	72-yr-old woman; myelodysplasic syndrom; (diabetic) nodular glomerulosclerosis (no clinical history of diabetes mellitus)	nesidioblastosis	hyperinsulinemic hypoglycemia	post mortem analysis	potential reversal of undiagnosed diabetes mellitus type II*	
135	Hashimoto et al.	1999	1		nesidioblastosis	hyperinsulinemic hypoglycemia	distal pancreatectomy		
136	Habanec et al.	1999	?	large atuopsy study	adult nesidioblastosis			unclear, how many (adult) cases*	
137	Masayuki et al.	1999	1		nesidioblastosis			Cited in Maeda, 2013*	
138	Espinosa-de-los-Montreros et al.	1999	1	coexistence of islet cell adenomatosis; nesidioblastosis; hyperplasia of islets and type 2 diabetes	nesidioblastosis	hyperinsulinemic hypoglycemia		Cited in Wong, 2019*	
139	Thompson et al.	2000	5	all with severe postprandial hypoglycemia, 29-64-yr-old; 3x male, 2x female	islet cell hypertrophy/nesidioblastosis	hyperinsulinemic hypoglycemia	distal pancreatectomy		
140	Kim et al.	2000	1	72-yr-old man; several syncopes	diffuse islet cell hyperplasia without nesidioblastosis	hyperinsulinemic hypoglycemia	partial pancreatectomy		
141	van der Wal et al.	2000	2	16- and 55-yr-old women	focally increased islet tissue, hypertrophic beta cells	hyperinsulinemic hypoglycemia	Whipple/distal pancreatectomy		
142	White et al.	2000	1	concomitant pancreatic endocrine microadenoma	nesidioblastosis	hyperinsulinemic hypoglycemia		*	
143	Kon et al.	2000	1	57-yr-old woman; type 2 diabetes	islet cell hyperplasia; nesidioblastosis	hyperinsulinemic hypoglycemia	85% pancreatectomy	*	
144	Hellman et al.	2000	3	report of 65 patients, including 3 with nesidioblastosis, age 15-89 years of the total sampe; 1 patient with concomitant insulinoma	nesidioblastosis	hyperinsulinemic hypoglycemia	partial pancreatectomy	1 patient possibly insulinoma with background nesidioblastosis	
145	Culberson et al.	2001	?	large autopsy study with children and adults suffering from sickle cell disease	nesidioblastosis, islet cell dispersion/hyperplasia/hypertrophy			unclear, how many (adult) cases*	
146	Zhao et al.	2001	1	51-yr-old man; coexisting intraductal papillary mucinous hyperplasia (IPMH) and (non-functioning?) islet cell tumor	nesidioblastosis	hyperinsulinemic hypoglycemia	distal pancreatectomy		
147	Sumarac-Dumanovic et al.	2001	2	41-yr-old female; 53-yr-old female; both with positive fasting test	islet cell hyperplasia and nesidioblastosis	hyperinsulinemic hypoglycemia	70-75% distal pancreatectomy; one case with completion pancreatectomy		
148	Burmeister et al.	2001	?		nesidioblastosis			*	Cited in Jabri, 2004
149	Witteles et al.	2001	5	22- to 48-yr-old (3x women, 2x men)	nesidioblastosis, hyperchromatic cell nuclei	hyperinsulinemic hypoglycemia	all 70% distal pancreatectomy; 2 patients need further treatment with Ca-channel blocker (Verapamil)		
150	Lecube et al.	2001	1	female	nesidioblastosis	hyperinsulinemic hypoglycemia		*	
151	Govindarajan et al.	2001	3	study on fibrocalculous pancreatic diabetes, 21- to 50-yr-old	nesidioblastosis; some with decrease in number of islets; some with signs of hyperplasia			"regenerative" type of nesidioblastosis as seen in cystic fibrosis/chronic pancreatitis?	
152	Meckler et al.	2001	11	family with fibrocystic pancreatic atrophy and pancreatic carcinoma	endocrine cell hyperplasia; nesidioblastosis-like pattern		all total pancreatectomy	possibly secondary to the fibrocystic atrophy	
153	Espinosa-de-los-Montreros et al.	2001	1	Patient with FAP (familial adenomatous polyposis coli)	nesidioblasotis	hyperinsulinemic hypoglycemia	distal pancreatectom	*	
154	Christesen et al.	2002	1	42-yr-old woman; activating Glucokinase mutation		relative hyperinsulinemia			
155	Semakula et al.	2002	1	35-yr-old woman; had pancreas transplantation for diabetes mellitus type I; hyperprolactinemia	nesidioblastosis; increased number of islets; nesidiodysplasia (large, hyperchromatic cell nuclei); scattered islet cell aggregates	hyperinsulinemic hypoglycemia	complete allograft removal		
156	Matthews et al.	2002	1	concomitant insulinoma	diffuse nesidioblastosis	hypoglycemia	partial pancreatectomy	*	
157	Carneiro et al.	2002	1		nesidioblastosis	hyperinsulinemic hypoglycemia	partial pancreatectomy		
158	Casas Vara et al.	2002	?		focal and diffuse nesidioblastosis			*	Cited in Jabri, 2004
159	Lu et al.	2002	1	hypopituitarism, secondary adrenal insufficiency; 84-yr-old woman; orbital lymphoma; fasting test negative	nesidioblastosis	hyperinsulinemic hypoglycemia	80% pancreatectomy	*	
160	Gloyn et al.	2003	2	20-yr-old woman (presenting with seizures); 1 adult man (Diagnosed after diagnosis of the son); both with proven activating glucokinase mutations	no nesidiobalstosis in the woman; no histology available from the man	hyperinsulinemic hypoglycemia (only in the woman)	pancreatic head resection + diazoxide (only the woman)		
161	Kaczirek et al.	2003	5	4 female, 1 male; 29- to 59-yr-old; one patient with additional multiple small insulinomas (islet cell adenomatosis); case-series of 66 cases with adult organic hyperinsulinism	islet hypertrophy; pleomorphic ß-cell nuclei, ductuloinsular complexes	hyperinsulinemic hypoglycemia	distal pancreatectomy		
162	Vella et al.	2003	8	clinically defined Non-insulinoma pancreatogenous hypoglycemia				*	
163	Branco et al.	2003	1	29-yr-old, negative fasting test	nesidioblastosis	hyperinsulinemic hypoglycemia	distal pancreatectomy	*	
164	Martignoni et al.	2003	1	54-yr-old man; oral antidiabetics for suspected diabetes mellitus type II	Glucagon-reactive nesidioblastosis; increased islets; ductulo-insular complexes; A-cells also in the center of the islets; Ki-67 < 1%		duodenopancreatectomy	perhaps secondary hypoglycemia due to glycogen-storage depletion in response to hyperglucagonemia	
165	Giorgadze et al.	2004	1	49-yr-old woman; hypertension, insulin-dependent diabetes mellitus, Hashimoto thyroiditis, chronic renal failure, renal stones; incidental left adrenal mass (adenoma); ovarian thecal metaplasia	nesidiodysplasia in pancreas	unclear	resection	unclear significance of the (incidental) detection of nesidiodysplasia of the pancrease	
166	Ito et al.	2004	1	84-yr-old woman; repeated unconsciousness	islet cell hyperplasia	hyperinsulinemic hypoglycemia	distal pancreatectomy (two step)	*	
167	Wiesli et al.	2004	2		islet cell hyperplasia	hyperinsulinemic hypoglycemia		unclear, if all adult* (Journal: Clinical Endocrinology)	
168	Wiesli et al.	2004	2		islet cell hyperplasia	hyperinsulinemic hypoglycemia		unclear, if same patients as in Wiesli et al. 2004, Clinical Endocrinology); here: Journal of Vascular and Interventional Radiology	
169	Proye et al.	2004	2	patients with MEN1; both with insulinoma	nesidioblastosis	hyperinsulinemic hypoglycemia	resection		
170	Kaczirek et al.	2004	4	series of 67 cases with adult hyperinsulinemic hypoglycemia; 4 matching the criteria of nesidioblastosis	nesidioblastosis	hyperinsulinemic hypoglycemia	resection	*	
171	Boulanger et al.	2004	1	36-yr-old woman; treated with octreotide through pregnancy; in coure of pregnancy decrease in octreotide need (physiological insulin resistance despite hyperinsulinism); normal infant	nesidioblastosis	hyperinsulinemic hypoglycemia	infusion of octreotide	caesarean section; normal development of child without any signs of malformation	
172	Chetty et al.	2004	1	41-yr-old woman; von Hippel-Lindau disease; bilateral pheochromocytoma; renal cell carcinoma; multiple lesions in the pancreas with islet hyperplasia, microadenomas, nesidioblastosis and an endocrine carcinoma	islet cell hyperplasia, nesidioblastosis	not symptomatic	total pancreatectomy	potential overlap with MEN1?	
173	Anlauf et al.	2005	15	study of 232 patients with persistent hyperinsulinemic hypoglycemia; 15 with nesidioblastosis	nesidioblastosis; ß-cell hypertrophy; enlarged and hyperchromatic ß-cell nuclei	hyperinsulinemic hypoglycemia	surgery	*	

174	Patti et al.	2005	3	27- 66-yr-old; 2x female, 1x male; all with gastric bypass (2x Roux-en-Y; 1x vertical banded gastroplasty)	diffuse islet hyperplasia and increased beta cell mass	severe postprandial, hyperinsulinemic hypoglycemia unrepsponsive to dietary changes/octreotide/diazoxide	partial pancreatectomy	to the best of our knowledge first definite report of post-bariatric surgery nesidioblastosis	
175	Pedrazzoli et al.	2005	4		islett hyperplasia/nesidioblastosis	hyperinsulinemic hypoglycemia	partial pancreatectomy	unclear, if all adult*	
176	Service et al.	2005	13	5 women, 1 man: 39- to 54-yr-old; all had previous Roux-en-Y gastric bypass; 7 additional cases with adult nesidioblastosis without gastric bypass were diagnosed in the same institution during the reported period (rest reported in Service, 1999 and Thompson, 2000)	nesidioblastosis	hyperinsulinemic hypoglycemia	partial pancreatectomy	post-bariatric surgery nesidioblastosis	
177	Babinska et al.	2005	1	48-yr-old woman	nesidioblastosis	hyperinsulinemic hypoglycemia	subtotal distal pancreatectomy + completion pancreatectomy	*	
178	Chen et al.	2005	1	45-yr-old man; necrolytic migratory erythema; mild diabetes	diffuse A cell hyperplasia	mild diabetes		*	
179	Tsujino et al.	2005	1	78-yr-old man; diagnosis with selective arterial calcium infusion	nesidioblastosis, diffuse islet cell hyperplasia	hyperinsulinemic hypoglycemia	60% distal pancreatectomy		
180	Kondo et al.	2005	1	72-yr-old woman; unclear hyperinsulinism that persisted after partial pancreatectomy		hyperisnulinemic hypoglycemia	partial pancreatectomy + additional treatment with long-acting somatostatin	unclear, if really nesidioblastosis	
181	Won et al.	2006	10	9x men, 1x woman; 29- to 78-yr-old; all with noninsulinoma pancreatogenous hypoglycemia with postprandial neuroglycopaenia and negative fasting tests	abnormal expression of INGAP (islet neogenesis associated protein)	hyperinsulinemic hypoglycemia	6x distal pancreatic resection; 4x diazoxide treatment		
182	Diaz et al.	2006	1		nesidioblastosis	hyperinsulinemic hypoglycemia	surgery	*	
183	Raffel et al.	2006	4	one of the patients had diabetes mellitus type II before; conversion to persistent hyperinsulinemic hypoglycemia (40-yr-old man)	enlarged and hyperchromatic nuclei; enlarged islet; cytologic abnormalities only in ß-cells	hyperinsulinemic hypoglycemia	surgery		
184	Starke et al.	2006	11	18- to 66-yr-old; mostly non-obese (when compared to insulinoma); 9x female, 2x male	islet hyperplasia, hypertrophy, microadenomatosis	hyperinsulinemic hypoglycemia	surgery		
185	Meier et al.	2006	4	all patients with gastrinoma/Zollinger-Ellison syndrom; 45- to 57-yr-old	fractional beta cell area, islet size and replication rate of beta cells was increased adjacent to the gastrinomas (but not > 1 cm away)		surgery		
186	Wiesli et al.	2006	1 (2)	2 women with HIV infection and narcotic addicts; no retroviral therapy	nesidioblastosis (histologically condirmed only in 1 patient)	hyperinsulinemic hypoglycemia	distal pancreatectomy (one of the patients)	*	
187	Arao et al.	2006	1	54-yr-old man; impaired glucose tolerance; fasting hypoglycemia	hypertrophy of islets; ductulo-insular complexes	hyperinsulinemic hypoglycemia	subtotal pancreatectomy + subsequent diazoxide		
188	Clancy et al.	2006	2	both post-gastric bypass patients	nesidioblastosis	hyperinsulinemic hypoglycemia	95% pancreatectomy (1x) + total pancreatectomy (in two steps)	post-bariatric surgery nesidioblastosis*	
189	Albazaz et al.	2006	1	76-yr-old man; pseudo-obstruction of bowels; microadenomas pancreas	islet cell hyperplasia (PP expressing cells)		duodenopancreatectomy		
190	Raffel et al.	2006	4	most likely the same collective as reported by Raffel et al. 2006 (18 to 58-yr-old; 3x men, 1x woman)	nesidioblastosis	hyperinsulinemic hypoglycemia	distal pancreatectomy		
191	Nakagawa et al.	2007	1	40-yr-old man; Billroth-II-Braun-gastrectomy approx. 4 years before symptom onset (+ Arnold-Chiari malformation, Klippel-Feil syndrome, recurrent duodenal ulcers)	nesidioblastosis	hyperisnulinemic hypoglycemia	distal pancreatectomy	post-bariatric surgery nesidioblastosis	
192	Alvarez et al.	2007	1	34-yr-old woman; Roux-en-Y gastric bypass 12 months before symptom onset	nesidioblastosis	hyperinsulinemic hypoglycemia	distal pancreatectomy	post-bariatric surgery nesidioblastosis	
193	Tseng et al.	2007	5	45- to 78-yr-old (2x men, 3x women)	nesidioblastosis	hyperinsulinemic hypoglycemia	40-70% distal pancreatectomy		
194	Bantle et al.	2007	3	1 women, 2 men; 50- to 65-yr-old; clinical diagnosis		hyperinsulinemic hypoglycemia	diet modification	possibly post-bariatric surgery nesidioblastosis	
195	Sahloul et al.	2007	1	78-yr-old man	increased number of islets + ectopic islets; proliferation of A, B, and D cells	hyperinsulinemic hypoglycemia	70% pancreatectomy	*	
196	Costa et al.	2007	1	34-yr-old	nesidioblastosis	hyperinsulinemic hypoglycemia	distal pancreatectomy		
197	Rosman et al.	2007	1	34-yr-old man; metastatic insulinoma (11 years after enucleation of insulinoma; 5 years after distal pancreatectomy for nesidioblastosis)	nesidioblastosis	hyperinsulinemic hypoglycemia	distal pancreatectomy + radiofrequency ablation of liver metastases	possibly insulinoma with background nesidioblastose	
198	Kok et al.	2007	1	24-yr-old woman with jejunal, heterotopic pancreas causing intussusception	nesidioblastosis	only abdominal pain	resection		
199	Kauhanen et al.	2007	2	34-yr-old woman; 63-yr-old man	nesidioblastosis (2x focal nesidioblastosis with corresponding uptake in 18F-DOPA PET)	hyperinsulinemic hypoglycemia	subtotal pancreatectomy		
200	Vezzosi et al.	2007	1		nesidioblastosis	hyperinsulinemic hypoglycemia	resection		
201	Bunning et al.	2007	1	71-yr-old man	islet cell hyperplasia (PP expressing cells)		duodenopancreatectomy		
202	Hong et al.	2008	1	71-yr-old man; subtotal gastrectomy two years before symptom onset	nesidioblastosis, hyperchromatic cell nuclei	hyperinsulinemic hypoglycemia	distal pancreatectomy	post-gastric surgery nesidioblastosis	
203	Yu et al.	2008	1	60-yr-old woman; nesidioblastosis, hypeprlasia of A cells, microglucagonoma and nonfunctioning islet cell tumor; concomitant meningioma	nesidioblastosis and hyperplasia of α cells	elevated glucagon, but now glucagonoma syndrome	resection (pylorus-sparing pancreaticoduodenectomy) + somatostatin analogues		
204	Moreira et al.	2008	1	26-yr-old woman; Roux-en-Y bypass approx. 16 months before symptom onset; NIPHS suspected clinically; no fasting hypoglycemia but postprandial symptomsr			Verapamil + Acarbose	post-bariatric surgery nesidioblastosis	
205	Kovacs et al.	2008	2	34-yr-old female, 22-yr-old male; both with positive fasting test; nesidioblastosis suspected clinically		hyperinsulinemic hypoglycemia	Diazoxid	*	
206	Abellan et al.	2008	1	51-yr-old man; symptom onset 6 months after bariatric surgery; insulinoma in pancreatic tail	nesidioblastosis with hypertrophy/hyperplasia of islets	hyperinsulinemic hypoglycemia	resection	post-bariatric surgery nesidioblastosis	
207	Kellogg et al.	2008	12	12 patients from a cohort of 14 post-bypass surgery patients exhibited clinical symptoms of hypoglycemia and showed hyperinsulinemic hypoglycemia		hyperinsulinemic hypoglycemia	Acarbose + low carbohydrate diet	post-bariatric surgery nesidioblastosis	
208	Dissanayake et al.	2008	1	38-yr-old woman; psychotic behaviour; concomitant insulinoma	nesidioblastosis	hyperinsulinemic hypoglycemia	subtotal pancreatectomy		
209	Z´graggen et al.	2008	2	cohort of 12 cases; 27- to 49-yr-old; 2 men, 10 women after Roux-en-Y bypass with severe hypoglycemia (insulin/C-Peptide not determined); clinical diagnosis	nesidioblastois confirmed in 2 cases pathologically		subtotal pancreatectomy in 2 cases; restoration of gastric restriction in the remaining cases	possibly post-bariatric surgery nesidioblastosis	
210	Karawagh et al.	2008	1		nesidioblastosis	hyperinsulinemic hypoglycemia	distal pancreatectomy	*	
211	Barbour et al.	2008	2	after Roux-en-Y bypass	islet cell hyperplasia		surgery	post-bariatric surgery nesidioblastosis*	
212	Kenney et al.	2008	1	45-yr-old woman; oxycodone abuse; seizures	diffuse islet cell hyperplasa	hyperinsulinemic hypoglycemia	subtotal pancreatectomy		
213	Geraghty et al.	2008	1	50-yr-old man, morning sweats, tremor	diffuse islet cell hyperplasia, nesidioblastosis	hyperinsulinemic hypoglycemia	distl pancreatectomy		
214	Toyomasu et al.	2008	1	71-yr-old man; subtotal gastrectomy	diffuse nesidioblastosis	hyperinsulinemic hypoglycemia	distal pancreatectomy	potentially not post-gastric surgery since no postprandial hypoglycemia	
215	Catton et al.	2008	1	66-yr-old man; hypogylcemia 2 years after esophagectomy; foreign-body granuloma above pancreatic tail	islet cell hyperplasia; nesidioblastosis	hyperinsulinemic hypoglycemia	distal pancreatectomy		
216	Wig et al.	2008	1	20-yr-old man; recurrent abdominal pain; vomitins (since age of 5); chronic familial pancreatitis	nesidioblastosis	hyperinsulinemic hypoglycemia		*	
217	Bright et al.	2008	1	35-yr-old female; positive fasting test; concomitant insulinoma	nesidioblastosis	hyperinsulinemic hypoglycemia	surgery		
218	Restrepo et al.	2009	?	concomitant insulinoma	nesidioblastosis			*	Cited in Garcia-Santos, 2013
219	Zhao et al.	2009	1		islet cell hyperplasia	hyperinsulinemic hypoglycemia	surgery	*	
220	Dong et al.	2009	1	84-yr-old male; diabetes mellitus type II (with insulin therapy); fasting hypoglycemia	islet cell hyperplasia, nesidioblastosis	hyperinsulinemic hypoglycemia	70% distal pancreatectomy	*	
221	Kaiser et al.	2009	?	post-gastric bypass		hyperinsulinemic hypoglycemia		*	
222	Andronesi et al.	2009	2		nesidioblastosis	hyperinsulinemic hypoglycemia	resection	unclear, if adult*	
223	Ahn et al.	2009	1	29-yr-old woman; primigravida; gestational diabetes; hypoglycemia after delivery; diabetes after resection	nesidioblastosis	hyperinsulinemic hypoglycemia	95% pancreatectomy		
224	Toyomasu et al.	2009	1	82-yr-old man; hypoglycemic syncope in the early morning	diffuse nesidioblastosis	hyperinsulinemic hypoglycemia	distal pancreatectomy	*	
225	Rumilla et al.	2009	>=23	27 cases of post-bariatric bypass nesidioblastosis (25 women; 2 men); 9 cases of idiopathic nesidioblastosis in adults (3 women, 6 men); subset already reported in Service, 2005	nesidioblastosis; 50% associated with peliosis-like vascular ectasia	hyperinsulinemic hypoglycemia	distal pancreatectomy		
226	Francesconi et al.	2009	1	60-yr-old man; fecal urgency, diarrhea; focal uptake in 111In-Pentetreotide scan	nesidioblastosis	no signs of hyperinsulinism	Whipple´s procedure	unclear, if occult VIPoma	
227	Thaler et al.	2009	135	discussion of approx. 135 cases of post-bariatric surgery (Roux-en-Y) hyperinsulinemic hypoglycemia on a conference; unclear, how many of them have been reported before	nesidioblastosis		some with distal pancreatectomy	unclear, which cases have been reported before	
228	Henopp et al.	2009	4	25- to 44-yr-old (2x men, 2x women); no association with VHL and MEN1	islett cell hyperplasia (A cell excess)		autopsy/pancreatic resection		
229	Spanakis et al.	2009	1	52-yr-old woman; post-gastric bypass; clinical diagnosis		hyperisnulinemic hypoglycemia	diazoxide	possibly post-bariatric surgery nesidioblastosis	
230	Aasheim et al.	2009	1	41-yr-old woman; after duodenal switch		hyperinsulinemic hypoglycemia		possibly post-bariatric surgery nesidioblastosis*	

231	Halperin et al.	2010	1	59-yr-old woman; Roux-en-Y-bypass		hyperinsulinemic hypoglycemia	glucagon	possibly post-bariatric surgery nesidioblastosis	
232	Mengual et al.	2010	?		nesidioblastosis			*	Cited in Garcia-Santos, 2013
233	Guseva et al.	2010	1	65-yr-old woman; post-gastric bypass surgery (26 years earlier); postprandial hypoglycemia; intraductal papillary mucinous tumor (stable); selective arterial calcium stimulation suggestive of nesidioblastosis	only clinical	hyperinsulinemic hypoglycemia	nifedipine treatment (30 mg/day)	post-bariatric surgery nesidioblastosis	
234	Moreno Moreno et al.	2010	3		nesidioblastosis	hyperinsulinemic hypoglycemia	surgery	unclear, if adult*	
235	Goretzki et al.	2010	16	13- to 89-yr-old; some of them may have been reported before; declared as noninsulinoma pancreatogenous hypoglycemia syndrome	nesidioblastosis	hyperinsulinemic hypoglycemia	surgery	unlear, how many reported before by the authors	
236	Ballester et al.	2010	1	female	nesidioblastosis	hyperinsulinemic hypoglycemia		*	
237	Bränström et al.	2010	1	35-yr-old man; electrophysiological studies	nesidioblastosis	hypeinsulinemic hypoglycemia	80% pancreatectomy		
238	McElroy et al.	2010	1	44-yr-old man	focal nesidioblastosis (nesidioblastoma); exophytic lesion; variably enlarged islets; clear cytoplasm, anisonucleosis; hyperchromatic nuclei; prominent nucleoli; larger islets + higher islet density; ß-cells according to immunohistochemistry; no increased Ki-67 positivity; distribution of somatostatin, glucagon and pancreatic polypeptide similar to controls	hyperinsulinemic hypoglycemia	enucleation (less than 5% of pancreas)		
239	Kibebew et al.	2010	1		nesidioblastosis	hyperinsulinemic hypoglycemia	partial pancreatectomy	unclear, if really adult*	
240	Mathavan et al.	2010	9	post-gastric bypass patients; 28- to 62-yr-old; 8x women; 1x male; symptom onset 1 to 56 months after bypass surgery	compatible with nesidioblastosis	hyperinsulinemic hypoglycemia	extended distal pancreatectomy	post-bariatric surgery nesidioblastosis	
241	Reubi et al.	2010	7	study on GLP-1 expression in tissue samples from 7 patients with post-gastric bypass (all Roux-en-Y) hyperinsulinemic hypoglycemia; 28- 69-yr-old; 6x women, 1x men	compatible with nesidioblastosis	hyperinsulinemic hypoglycemia	partial pancreatectomy	post-bariatric surgery nesidioblastosis	
242	Vanderveen et al.	2010	75	48 completed survey (13 men, 35 women; 15- to 78-yr-old); non-respondents (21; 28- to 63-yr-old; 6 men, 15 women) + 5 deaths + 1 in prison; from 75: 48 had prior bariatric surgery; some also gastrectomy/esophagectomy/anti-ulcer or anti-reflux procedure	nesidioblastosis/islet cell hyperplasia	hyperinsulinemic hypoglycemia	mostly distal pancreatectomy	many post-bariatric surgery patients; unclear, if any of the cases have been reported before in the literature	
243	Bernard et al.	2010	1	69-yr-old man; prior fundoplication surgery; presents with postprandial hypoglycemia and positive selective arterial calcium stimultion; declined surgery		hyperinsulinemic hypoglycemia	octreotide treatment	possibly case of post-surgery nesidioblastosis	
244	Rabiee et al.	2011	1	47-yr-old woman, post-gastric bypass	nesidioblastosis/islet cell hyperplasia	hyperinsulinemic hypoglycemia	85% pancreatectomy	post-gastric surgery nesidioblastosis	
245	Arafah et al.	2011	1	16-yr-old female; concomitant pseudopapillary neoplasm of the pancreas	islet cell hyperplasia	hypoglycemia	distal pancreatectomy	*	
246	Nayak et al.	2011	1	45-yr-old woman; lesion in the uncinate process	focal islet cell hyperplasia	hyperinsulinemic hypoglycemia	enucleation		
247	Batra et al.	2011	1	55-yr-old woman; symptoms not related to fasting	nesidioblastosis	hyperinsulinemic hypoglycemia	distal pancreatectomy	*	
248	Otto et al.	2011	1	44-yr-old man; glucagonoma syndrome	diffuse A cell hyperplasia		octreotide treatment		
249	Azemoto et al.	2012	1	59-yr-old woman; no glucagonoma syndrome	A cell hyperplasia		distal pancreatectomy		
250	Roberts et al.	2012	1	56-yr-old male; glucagonoma with GLP-1 secretion (metastatic); first diabetes, later hypoglycemia	islet cell hyperplasia of the pancreas	hyperinsulinemic hypoglycemia	surgery		
251	Qintar et al.	2012	1	40-yr-old female; Roux-en-Y bypass 6 years before presentation	diffuse islet cell hypertrophy, pleomorphism, ductulo-insular complexes	hyperinsulinemic hypoglycemia	80% pancreatectomy	post-bariatric surgery nesidioblastosis	
252	Limongelli et al.	2012	1	27-yr-old woman; morbidly obese	islet cell hyperplasia	hyperinsulinemic hypoglycemia	distal pancreatectomy		
253	De Heide et al.	2012	1	25-yr-old woman; post-Roux-en-Y bypass; clinical diagnosis of hyperinsulinemic hypoglycemia		hyperinsulinemic hypoglycemia	laparoscopic adjustable banding for pouch dilatation	possibly post-bariatric surgery nesidioblastosis	
254	Myint et al.	2012	1	42-yr-old woman; 6 years after gastric bypass surgery		hyperinsulinemic hypoglycemia	octreotide treatment	possibly post-bariatric surgery nesidioblastosis	
255	Nadelson et al.	2012	1	51-yr-old woman after Roux-en-Y gastric bypass (multiple revisions; gastrectomy)		hyperinsulinemic hypoglycemia	acarbose	possibly post-bariatric surgery nesidioblastosis	
256	Przybylik-Mazurek et al.	2012	5	adult; 3 with concomitant small insulinomas	islet cell hyperplasia	hyperinsulinemic hypoglycemia	distal pancreatectomy	*	
257	Ceppa et al.	2012	?	Post-gastric-bypass	nesidioblastosis	hyperinsulinemic hypoglycemia		*	
258	Ferrario et al.	2012	1	23-yr-old man	nesidioblastosis	hyperinsulinemic hypoglycemia	duodenopancreatectomy + Diazoxide		
259	Lee et al.	2013	2	35- and 62-yr-old women after Roux-en-Y bypass; clinical diagnosis		hyperinsulinemic hypoglycemia	bypass reversal	possibly post bariatric surgery nesidioblastosis	
260	Christ et al.	2013	3	Detection via 111In-DTPA-exendin-4 SPECT/CT (2x false positive, 1x true negative); study on insulinoma detection	islet cell hyperplasia	hyperinsulinemic hypoglycemia	surgery		
261	Soares et al.	2013	1	22-yr-old woman; obese, hypertensive; reccurent transient loss of consciousness (no relation to fasting); sinus tachycardia during events	islet cell hyperplasia and hypertrophy; ductulo-insular complexes; nesidiobalstosis	hyperinsulinemic hypoglycemia	total pancreatectomy (reccurent symptoms after distal pancreatectomy)		
262	Garcia-Santos et al.	2013	1	38-yr-old woman (previous duodenal ulcers with pyloroplasty and truncal vagotomy); later antrectomy + Roux-en-Y procedure	nesidioblastosis	hyperinsulinemic hypoglycemia	80% pancreatectomy		
263	Then et al.	2013	1	58-yr-old woman	islet cell hyperplasia	hyperinsulinemic hypoglycemia	repeated pancreatic resections up to total pancreatectomy		
264	Al-Sarireh et al.	2013	1	56-yr-old man; renal cell carcinoma; cystic lesion in pancreas	islet cell hyperplasia (A cell excess)		resection		
265	Choi et al.	2013	1	43-yr-old woman; comcomitant non-functioning pancreatic neuroendocrine tumor; type II diabetes	islet cell hyperplasia; hyperchromatic nuclei and pleomorphism, diffuse nesidioblastosis	hyperglycemia	duodenopancreatectomy		
266	Gupta et al.	2013	1	50-yr-old woman	nesidioblastosis	hyperinsulinemic hypoglycemia	subtotal pancreatectomy		
267	Sowa-Staszcak et al.	2013	1	38-yr-old woman; concomitant insulinoma	nesidioblastosis	hyperinsulinemic hypoglycemia	multiple pancreatic operations		
268	Pongpasobchai et al.	2013	2		nesidioblastosis	hyperinsulinemic hypoglycemia		unclear, if adult*	
269	Maeda et al.	2013	1	32-yr-old man	nesidioblastosis	hyperinsulinemic hypoglycemia	distal pancreatectomy		
270	Grönberg et al.	2013	1		nesidioblastosis			unclear if adult	
271	Challis et al.	2014	5	family with hyperinsulinemic hypoglycemia, all adult; activating GCK (Glukokinase) mutations, 33- to 84-yr-old	not available	hyperinsulinemic hypoglycemia	diazoxide/diet		
272	De Heide et al.	2014	1	post Roux-en-Y bypass		hyperinsulinemic hypoglycemia	pasireotide	Cited in Schwertz, 2016*; possibly post-bariatric surgery nesidioblastosis	
273	Campos et al.	2014	5	all post Roux-en-Y bypass; clinical hyperinsulinemic hypoglycemia; 38- to 49-yr-old; 4x women, 1x man		hyperinsulinemic hypoglycemia	bypass reversal (normal anatomy or modified sleeve gastrectomy)		
274	Kang et al.	2014	1	35-yr-old female; recurrent acute pancreatitis; glucagonoma; glucagon-producing microadenoma	diffuse hyperplasia of islets (A cells); alpha-cell nesidioblastosis		distal pancreatectomy	*	
275	Pathak et al.	2014	1	47-yr-old woman, type II diabetes, hypertension; Roux-en-Y bypass before; postprandial hypoglycemia	nesidioblastosis	hyperinsulinemic hypoglycemia	distal pancreatectomy	post-gastric surgery nesidioblastosis	
276	Rao et al.	2014	1	58-yr-old man; Roux-en-Y bypass nine months before symptoms		hyperinsulinemic hypoglycemia	bypass reversal		
277	de Santibanes et al.	2014	3		nesidioblastosis	hyperinsulinemic hypoglycemia	distal pancreatectomy	*	
278	Garcia et al.	2014	2	2x women (36-yr-old), post-gastric bypass	islet cell hyperplasia	hyperinsulinemic hypoglycemia	distal pancreatectomy	post-bariatric surgery nesidioblastosis	
279	Woo et al.	2015	5	5 women; 48- to 86-yr-old; no history of gastric bypass; one patient hat concomitant insulin autoantibodies	nesidioblastosis	hyperinsulinemic hypoglycemia	distal to subtotal pancreatectomy		
280	Martin-Grace et al.	2015	1	28-yr-old woman, exercise-induced hypoglycemia	islet cell hyperplasia, nesidioblastosis	hyperinsulinemic hypoglycemia	distal pancreatectomy + amlodipine		
281	Valli et al.	2015	1	59-yr-old woman; small neuroendocrine tumor in pancreatic tail (non-functioning)	nesidioblastosis	hyperinsulinemic hypoglycemia	distal pancreatectomy + amlodipine		
282	Ramirez-Gonzalez et al.	2015	1	46-yr-old woman; Sheehan syndrom	islet hyperplasia, diffuse nesidioblastosis	hyperinsulinemic hypoglycemia	distal pancreatectomy		
283	Thompson et al.	2015	74	cohort has been reported before in Vanderveen, 2010 (see above)	nesidioblastosis/islet cell hyperplasia	hyperinsulinemic hypoglycemia	distal pancreatectomy		
284	Maguire et al.	2015	1	34-yr-old man; concomitant neuroendocrine tumor	background islet cell microadenomatosis	hyperinsulinemic hypoglycemia	distal pancreatectomy		
285	Ünal et al.	2015	1	31-yr-old woman; history of gastric bypass surgery	nesidioblastosis	hyperinsulinemic hypoglycemia	distal pancreatectomy		
286	Fountoulakis et al.	2015	1	43-yr-old woman; disturbed counterregulatory hormone responses (impaired cortisol response); clinical diagnosis		hyperinsulinemic hypoglycemia	diazoxide		

287	Qin et al.	2015	1	62-yr-old man	focal, adult nesidioblastosis	hyperinsulinemic hypoglycemia	enucleation		
288	Sampaio-Neto et al.	2015	3	32- to 43-yr-old women; post-gastric bypass patients, clinical diagnosis		hyperinsulinemic hypoglycemia	bypass reversal		
289	Christ et al.	2015	1	65-yr-old woman (negative 18F-DOPA-PET/CT; 68Ga-DOTATATE-PET/CT and arterial calcium stimulation); diffuse uptake in 68Ga-DOTA-exendin-4 PET/CT	nesidioblastosis	hyperinsulinemic hypoglycemia	distal pancreatectomy		
290	Mordes et al.	2015	6	all patients after Roux-en-Y bypass; age 13- to 57-yr-old; clinical diagnoses		hyperinsulinemic hypoglycemia	diet/acarbose/calcium channel inhibitor; no pancreatectomy	possibly post-bariatric surgery nesidioblastosis	
291	Mihai et al.	2015	1	57-yr-old	nesidioblastosis; isolated cells and areas of focal distribution	hyperinsulinemic hypoglycemia	distal pancreatectomy		
292	Macedo et al.	2016	1	34-yr-old woman; post-Roux-en-Y bypass	nesidioblastosis	hyperinsulinemic hypoglycemia	subtotal pancreatectomy + bypass reversal to sleeve gastrectomy		
293	Kim et al.	2016	1	54-yr-old woman	focial hyperplasia/focal nesidioblastosis; nuclear hyperchromasia	hyperinsulinemic hypoglycemia	pylorus-preserving pancreatoduodenectomy	focal nesidioblastosis	
294	Yang et al.	2016	1	29-yr-old female	nesidioblastosis	hyperinsulinemic hypoglycemia	distal pancreatectomy		
295	Luo et al.	2016	4	clinical diagnosis; 2 at school age with proven activating mutations of glucokinase		hyperinsulinemic hypoglycemia		definite age unknown	
296	Schwetz et al.	2016	1	46-yr-old female; no hyperglucagonemia	islet cell hyerplasia; microadenoma with glucagon expression	hyperinsulinemic hypoglycemia	distal pancreatectomy + pasireotide		
297	Prasad et al.	2016	3	3x women; 61- to 67-yr-old	nesidioblastosis	hyperinsulinemic hypoglycemia	distal pancreatectomy or Lanreotide		
298	Krieger et al.	2016	5	2 cases with insulinoma + nesidioblastosis, 3 cases with nesidioblastosis alone	nesidioblastosis	hyperinsulinemic hypoglycemia	surgery	unclear, if all adult*	
299	Vilarrasa et al.	2016	2	post-gastric bypass surgey patients	nesidioblastosis	hyperinsulinemic hypoglycemia	partial pancreatectomy	post-bariatric surgery nesidioblastosis	
300	Sowa-Staszcak et al.	2016	4	2x combination of insulina with nesidioblastosis, 2x nesidioblastosis alone	nesidioblastosis	hyperinsulinemic hypoglycemia	distal pancreatectomy		
301	Gunnarsdottir et al.	2016	1	18-yr-old woman	nesidioblastosis	hyperinsulinemic hypoglycemia			
302	De Sousa et al.	2016	1	33-yr-old man; concomitant pancreatic neuroendocrine tumor (microadenoma), intraductal papillar mucinous neoplasia	nesidioblastosis (A and B cell hyperplasia)	hyperinsulinemic hypoglycemia (no clinics of glucagon excess)	subtotal pancreatectomy		
303	Anderson et al.	2016	1	67-yr-old man; COPD, hypertension, bradycardia (with transient complete heart block), alcohol abuse (Wernicke-Korsakoff syndrome)	nesidioblastosis/islet cell hyperplasia	hyperinsulinemic hypoglycemia	distal pancreatectomy + diazoxide and acarbose		
304	Preechasuk et al.	2016	1		nesidioblastosis	hyperinsulinemic hypoglycemia		*	
305	Chen et al.	2018	1	34-yr-old woman; post-Roux-en-Y bypass	nesidioblastosis; islet cell hyperplasia	hyperinsulinemic hypoglycemia	bypass reversal; 80% pancreatectomy (conservative treatment with diazoxide/octreotide/dietary modifications unsuccesful)	post-bariatric surgery nesidioblastosis	
306	Dadheech et al.	2018	1	28-yr-old male; post-Roux-en-Y bypass	nesidioblastosis; islet cell hyperplasia and significant proliferation	hyperinsulinemic hypoglycemia	distal pancreatectomy	post-bariatric surgery nesidioblastosis	
307	Antwi et al.	2018	1		nesidioblastosis (focal)	hyperinsulinemic hypoglycemia	surgery		
308	Luca et al.	2018			nesidioblastosis			patients already reported in Christ, 2013 and Antwi, 2018	
309	Kerekou et al.	2019	1	29-yr-old patient		hyperinsulinemic hypoglycemia	corticosteroid	possible case of adult nesidioblastosis	
310	Wiesli et al.	2019	1	76-yr-old man; malignant, metastasizing somatostatinoma with hyperglycemia and postprandial hypoglycemia	islet cell hyperplasia	hyperinsulinemic hypoglycemia	autopsy finding		
311	Lozano-Melendez et al.	2019	1	36-yr-old man; end stage chronic kidney disease (diagnosis with selective arterial calcium stimulation)	nesidioblastosis	hyperinsulinemic hypoglycemia	subtotal pancreatectomy		
312	Dauriz et al.	2019	1	63-yr-old woman; distal pancreatectomy for insulinoma 4 years ago; insulinoma relapse on background nesidioblastosis	nesidioblastosis; islet cell hyperplasia	hyperinsulinemic hypoglycemia	subtotal pancreatectomy (diazoxide and pasireotide did not work)	insulinoma with background nesidioblastosis	
313	Allue et al.	2019	1	male, middle-aged	nesidioblastosis			*	
314	Wong et al.	2019	1	67-yr-old woman; short bowel syndrome (secondary to accident); type 2 diabetes mellitus (managed with GLP-1 agonist)	nesidioblastosis	hyperinsulinemic hypoglycemia	distal pancreatectomy + octreotide (diazoxide and acarbose did not work due to short gut syndrome)		
315	Kim et al.	2019	1	60-yr-old woman after sleeve gastrectomy; cardiac conduction block	nesidioblastosis, islet cell hyperplasia	hyperinsulinemic hypoglycemia	total pancreatectomy		
316	Gouta et al.	2019	2	44-yr-old woman and 52-yr-old woman	islet cell hyperplasia	hyperinsulinemic hypoglycemia	caudal pancreatectomy/enucleation		
317	Orujov et al.	2019	1	49-yr-old woman; comcomitant neuroendocrine tumor (distal pancreatectomy); post-Roux-en-Y-bypass	nesidioblastosis	hyperinsulinemic hypoglycemia	distal pancreatectomy + completion pancreatectomy	possibly post-gastric bypass nesidioblastosis or insulinoma with background nesidioblastosis	
318	Delbeke et al.	2019	1	proven metastatic neuroendocrine tumor	islet cell hyperplasia (more focal)	not reported	distal pancreatectomy		
319	Kalff et al.	2020	3+4	2x post-gastric bypass; 1x without prior gastric surgery; additionally: 3 medically controlled post-gastric bypass surgery patients with diffuse enrichment and one case of suspected non-insulinoma pancreatogenous hypoglycemia syndrome with diffuse tracer enrichment	nesidioblastosis	hyperinsulinemic hypoglycemia	distal pancreatectomy; 1 relapse with completion pancreatectomy		
320	Yamada et al.	2020	111	Nationwide survey in Japan; 33 classified as post-gastric bypass (23 males, 10 females; aged 1 to 89 years) ; 57 classified as postprandial hyperinsulinemic hypoglycemia (21 males, 36 females; 8 month - 91 years); 10 classified as nesidioblastosis (5 men, 5 women; 19- to 79 years; only 6 surgically proven),	nesidioblastosis	hyperinsulinemic hypoglycemia	medical treatment or distal pancreatectomy in 6		
321	Lopes et al.	2020	1	32-yr-old man; concomitant with pancreatic heterotopia	nesidioblastosis + pancreatic heterotopia (and nesidioblastosis in the ectopic pancreatic tissue)	hyperinsulinemic hypoglycemia	distal pancreatectomy		
322	Antwi et al.	2020	1		nesidioblastosis	hyperinsulinemic hypoglycemia	distal pancreatectomy	unclear, if really adult	
323	Boss et al.	2020	3	clinically suspected cases of nesidioblastosis (surgically not confirmed)		hyperinsulinemic hypoglycemia	no surgery performed		
324	Awramiszyn-Fernandez et al.	2020	1	non-surgical weight loss	nesidioblastosis	hyperinsulinemic hypoglycemia		unclear, if really adult*	
325	McManus et al.	2020	1	45-yr-old woman; approximately 10 years after Roux-en-Y gastric bypass		hyperinsulinemic hypoglycemia		*	
326	Kato et al.	2021	1	73-yr-old man; on dialysis since 18 years	nesidioblastosis	hyperinsulinemic hypoglycemia	duodenopancreatectomy + continuous subcutaneous octreotide infusion + corticosteroids		
327	Snaith et al.	2021	1	40-yr-old woman; recurrent hypoglycemia after multiple insulinoma resections; no MEN1	islet cell hyperplasia/insulinomatosis/insulin-expressing monohormonal endocrine cell clusters	hyperonsulinemic hypoglycemia	total pancreatectomy with failure of cure		
328	Torres-Araono et al.	2021	1	31-yr-old		hyperinsulinemic hypoglycemia	surgery		
329	Castillo-López et al.	2022	1	15-yr-old man	diffuse β-cell hyperplasia	hyperinsulinemic hypoglycemia	subtotal pancreatectomy (failure of diazoxide		
330	Koneshamoorthy et al.	2022	1	22-yr-old man; obese; activating mutation in Glucokinase (also found in mother, sister, and nephew)	diffuse β-cell hyperplasia	hyperinsulinemic hypoglycemia	distal pancreatectomy; postoperative failure of verapamile, diazoxide, and octreotide; pasireotide successful	unclear, if really adult; first hypoglycemia episode as newborn	
331	Doi et al.	2022	1	55-yr-old woman	focal nesidioblastosis (mimicking insulinoma); hyperplastic nodular formation; ductulo-insular complexes	hyperinsulinemia	distal pancreatectomy		

